# Stomatal Function Requires Pectin De-methyl-esterification of the Guard Cell Wall

**DOI:** 10.1016/j.cub.2016.08.021

**Published:** 2016-11-07

**Authors:** Sam Amsbury, Lee Hunt, Nagat Elhaddad, Alice Baillie, Marjorie Lundgren, Yves Verhertbruggen, Henrik V. Scheller, J. Paul Knox, Andrew J. Fleming, Julie E. Gray

**Affiliations:** 1 Department of Animal and Plant Sciences, University of Sheffield, Sheffield S10 2TN, UK; 2 Department of Molecular Biology and Biotechnology, University of Sheffield, Sheffield S10 2TN, UK; 3 Department of Botany, University of Omar Al-Mukhtar, Al-Baida, Libya; 4 Biological Systems and Engineering Division and Joint BioEnergy Institute, Lawrence Berkeley National Laboratory, Berkeley, CA 94720, USA; 5 Centre for Plant Sciences, Faculty of Biological Sciences, University of Leeds, Leeds LS2 9JT, UK

**Keywords:** stomata, guard cell, cell wall, pectin, *Arabidopsis*

## Abstract

Stomatal opening and closure depends on changes in turgor pressure acting within guard cells to alter cell shape [1]. The extent of these shape changes is limited by the mechanical properties of the cells, which will be largely dependent on the structure of the cell walls. Although it has long been observed that guard cells are anisotropic due to differential thickening and the orientation of cellulose microfibrils [2], our understanding of the composition of the cell wall that allows them to undergo repeated swelling and deflation remains surprisingly poor. Here, we show that the walls of guard cells are rich in un-esterified pectins. We identify a pectin methylesterase gene, *PME6*, which is highly expressed in guard cells and required for stomatal function. *pme6-1* mutant guard cells have walls enriched in methyl-esterified pectin and show a decreased dynamic range in response to triggers of stomatal opening/closure, including elevated osmoticum, suggesting that abrogation of stomatal function reflects a mechanical change in the guard cell wall. Altered stomatal function leads to increased conductance and evaporative cooling, as well as decreased plant growth. The growth defect of the *pme6-1* mutant is rescued by maintaining the plants in elevated CO_2_, substantiating gas exchange analyses, indicating that the mutant stomata can bestow an improved assimilation rate. Restoration of *PME6* rescues guard cell wall pectin methyl-esterification status, stomatal function, and plant growth. Our results establish a link between gene expression in guard cells and their cell wall properties, with a corresponding effect on stomatal function and plant physiology.

## Highlights

• The guard cell wall is distinguished by a relatively low level of methylated pectin
• Increased methyl pectin leads to stomata with a smaller dynamic range of movement
• These plants show increased evaporative cooling and decreased growth under drought
• Elevated CO_2_ restores mutant plant growth to normal

## Graphical Abstract

**Figure:**
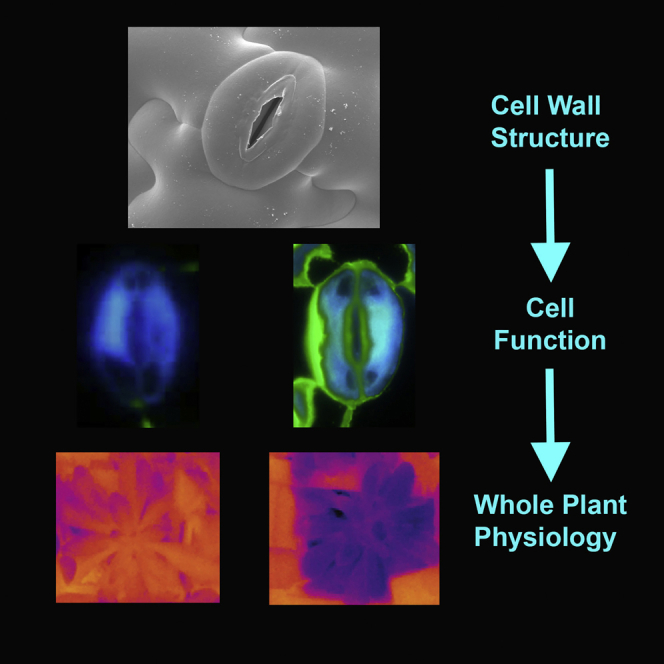

## Teaser

Guard cell wall mechanics must play a role in setting the dynamics of stomatal movement. Amsbury et al. show that the degree of pectin methylation in the wall sets the range of cell swelling, with consequences for plant water use and growth being dependent on CO_2_ level. Stomatal mechanics are likely to influence plant response to climate change.

## Results and Discussion

### Analysis of Guard Cell Wall Composition by an Antibody Screen

**Figure 1:**
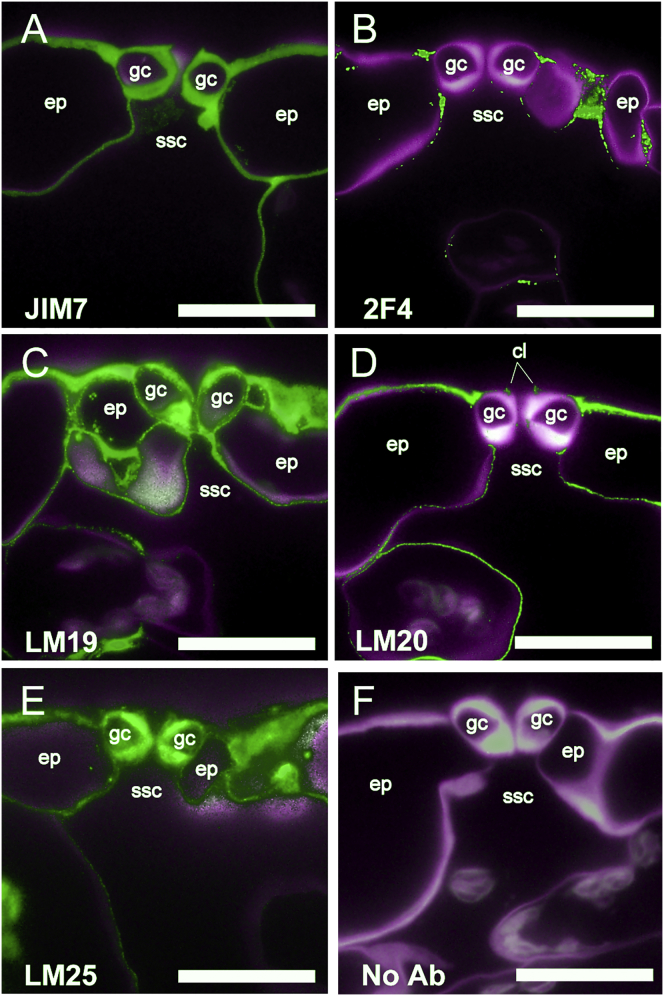
Guard Cells Show Specific Patterns of Wall Epitopes (A) Ubiquitous presence of pectin in cell walls. The JIM7 antibody binds to HGA with a broad range of methyl-esterification and shows labeling in all cell walls in a cross-section through the epidermis (ep) encompassing guard cells (gc) above a sub-stomatal cavity (ssc). (B) Calcium cross-linked HGA is restricted to cell interstices. The 2F4 antibody indicates cell walls containing calcium cross-linked HGA characterized by stretches of unesterified HGA residues. (C) Unesterified HGA is present in GC walls. Binding of the LM19 antibody indicates that HGA with no or little esterification is prevalent in all cell walls of the epidermis. (D) Highly methyl-esterified pectin is absent from the guard cell wall, as indicated by the lack of binding of the LM20 antibody. (E) Binding of the LM25 antibody indicates that xyloglucan is present in all cell walls of the epidermis. (F) A control with no primary antibody (Ab) showing low levels of autofluorescence against the Calcofluor White staining of the cell wall. In all panels, the green signal shows binding of the specific primary antibody indicated, and the magenta signal (false color) indicates Calcofluor White fluorescence of cell walls. Scale bars, 20 μm. See also Figure S1 and Table S1.

Probing *Arabidopsis thaliana* leaf sections with a panel of 36 monoclonal antibodies by fluorescence microscopy revealed a range of antibody-binding patterns, including clear differences in the composition of guard cell walls compared to epidermal or mesophyll cells (Figure 1; Figure S1; Table S1). Homogalacturonan (HGA) is a polysaccharide of α-1,4-linked galacturonic acid (GalA) residues and is the predominant form of pectin in *A. thaliana* [3, 4]. It is synthesized at the Golgi apparatus and secreted from cells in a highly methyl-esterified form. These methyl ester groups can subsequently be removed by enzymatic activity in the cell wall, allowing for a range of methyl-esterification states. A broad range of HGA methyl-esterification patterns are recognized by the JIM7 antibody [5], and Figure 1A shows uniform JIM7 binding within the walls of guard cells, epidermal pavement, and mesophyll cells, indicating a wide distribution of HGA. HGA can form pectate calcium cross-links when continuous stretches of GalA residues are blockwise de-esterified, and this can be detected using the 2F4 antibody [6]. De-esterified, calcium cross-linked HGA was not detected in the guard cell walls or in neighboring cell walls but, instead, was limited to the junction regions between cells of the epidermis (Figure 1B). In contrast, relatively unesterified HGA (detected by antibody LM19) showed strong labeling in guard cell and epidermal cell walls (Figure 1C). Highly methyl-esterified HGA, as indicated by antibody LM20, was excluded from guard cells but was abundant in the junctions between guard cells and neighboring epidermal cells (Figure 1D). The LM20 signal was observed in the cell walls of epidermal pavement and mesophyll cells, as well as the cuticular ledges of guard cells. This specific pattern of pectins contrasted with the more uniform signal observed following immunolocalization with antibodies against other cell wall components, such as xyloglucan (LM25) (Figure 1E), while signal was absent when the primary antibody was not included (Figure 1F). Out of 36 cell wall antibodies tested, LM20 (against high methyl-esterified HGA) revealed a clear guard-cell-specific pattern (Figure S1; Table S1) being absent from guard cell walls but detected strongly in neighboring cell walls.

These results can be compared with previous investigations of guard cell wall composition [7, 8]. For example, analysis of *Commelina communis* has shown that guard cells are rich in pectin but did not report on the differential methyl-esterification patterns described here. Our data show that both highly methyl-esterified and calcium cross-linked blockwise de-esterified HGA are excluded from the guard cells and that un-esterified HGA is the predominant form of pectin in the guard cell wall. Pectic arabinans have previously been implicated as being important for guard cell movements via ectopic application of carbohydrate-modifying enzymes [8]. Thus, our data support the idea that the structural properties of the pectin network are important for guard cell function. We took a molecular genetic approach to test this hypothesis.

### Identification of a Mutant, *pme6-1*, with Altered Guard Cell Wall Pectin Distribution

The enzymes that modify plant cell wall pectins are typically encoded by large gene families. For example 66, 35, and 89 pectin methylesterase genes have been annotated in *A. thaliana*, *Oryza sativa*, and *Populus trichocarpa,* respectively [9]. The encoded proteins contain pectin methylesterase (PME), or both PME and pectin methylesterase inhibitor domains (proPME proteins). The PME and proPME enzymes control the methyl-esterification status of HGA by removing methyl ester groups from HGA [9]. This large number of genes has made it difficult to attribute specific pectin modifications of cell walls to particular physiological properties. As our experiments identified guard cells as having a distinct pectin methyl-esterification status, we sought to identify genes encoding pectin-modifying enzymes with a guard-cell-specific expression pattern. We focused on a proPME gene, *PME6* (TAIR: *AT1G23200*), which is expressed at >36-fold higher levels in guard cell protoplasts relative to mesophyll cell protoplasts [10] and is also expressed during seed coat development [11]. *PME6* expression has previously been shown to be repressed in *scap1*, a mutant with altered expression of cell wall modification genes and a resultant change in methyl-esterification state [12]. Negi et al. [12] proposed that *PME6* might act downstream of *SCAP1* to elicit at least part of the stomatal phenotype observed.

*PME6* encodes a single PMEI domain and a PME domain that contains the two conserved active-site aspartic acid residues necessary for PME activity [9], as well as an N-terminal signal peptide, suggesting that it is a secreted protein. A *PME6* promoter β-glucuronidase (GUS) fusion construct containing approximately 1,400 bp upstream of the start codon (*proPME6::GUS*) was stably introduced into *A. thaliana*, and GUS histochemical localization indicated that this DNA region directs expression predominantly in mature guard cells (Figure S2A). Analysis of transcriptome data indicated that *PME6* mRNA accumulates to a high level in the *scrm-D* mutant, which has an excess of mature guard cells [13] and to a lower level in mutants in which epidermal cell differentiation is blocked at the pavement cell stage (*spch*) [14] or at the stage of meristemoid formation (*scrm-D mute*) [13, 15] (Figure S1B). These data suggest that *PME6* is expressed in guard cells at a relatively late stage of differentiation. Transcriptome data indicated that *PME6* is also expressed in siliques [16], and analysis of the *proPME6::GUS* lines confirmed this. Analysis of a *pme6* mutant (described later) did not reveal any change in seed germination or seed weight, so our further investigation focused on stomatal function. To investigate the function of *PME6*, we obtained an *A. thaliana* line with a Ds transposon inserted within the *PME6* gene (hereinafter referred to as *pme6-1*) from the Nottingham Arabidopsis Stock Centre. PCR and RT-PCR analyses showed that *pme6-1* homozygous plants harbor an insertion in the single intron of the *PME6* gene (Figure S2C) and have no detectable expression of the *PME6* mRNA transcript (Figure S2D). Complementation of the *pme6-1* line by introducing a native *PME6* gene construct under the control of the *proPME6* promoter restored the levels of *PME6* mRNA in two independent lines (*proPME6::pme6*) (Figure S2D).

**Figure 2:**
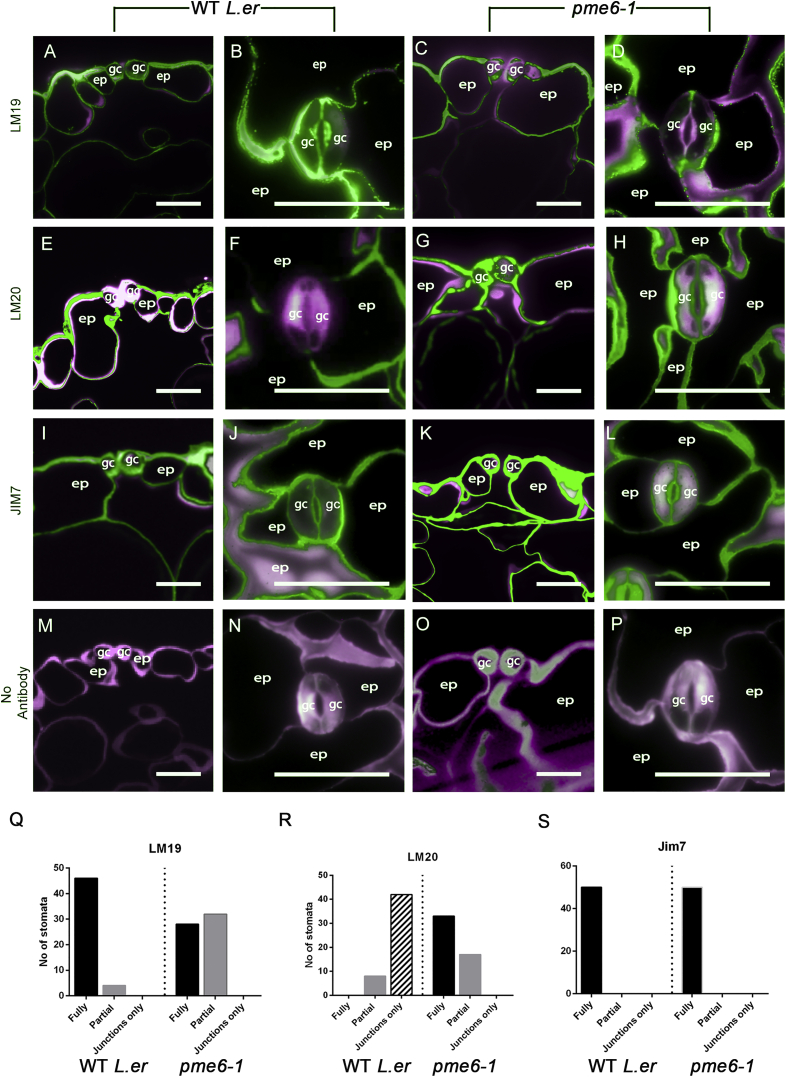
Guard Cell Wall Pectin Composition Is Altered in *pme6-1* Plants (A–D) The high level of unesterified HGA in WT guard cells indicated by LM19 antibody binding in both cross-sections (A) and paradermal sections (B) is greatly diminished in *pme6-1* (C and D). ep, epidermis; gc, guard cells. (E–H) Highly methyl-esterified HGA is absent in WT guard cell walls (E and F) but accumulates in the guard cell walls of the *pme6-1* mutant, as revealed by binding of the LM20 antibody (G and H). (I–L) The general distribution of HGA (indicated by the JIM7 antibody) is similar in the WT (I and J) and the *pme6-1* mutant (K and L). (M–P) Control sections not hybridized with primary antibody but stained with Calcofluor White indicate the signal specificity of the immunolabeling experiments in (A)–(L) and the general distribution of the cell wall material. In all panels, the green signal shows binding of the specific primary antibody indicated, and the magenta signal (false color) indicates Calcofluor White fluorescence of cell walls. (Q–S) Counting of stomata showing the patterns of labeling with each antibody indicate the switch in LM20/LM19 labeling pattern between WT and the *pme6-1* mutant guard cells. Localization of fluorescence in transverse sections after antibody binding was scored as fully covering guard cells (as in I), partially covering guard cells (as in C), or limited to guard cell-epidermal cell junctions (as in E). Data are shown for LM19 (Q), LM20 (R), and Jim7 (S) immunolabeling. Quantification was based on scoring patterns from 50 stomata, with five stomata scored from each of ten plants. Scale bars, 20 μm. See also Figures S1–S3.

Immunolocalization analyses of *pme6-1* revealed a major change in the methyl-esterification status of guard cell wall pectins. We first confirmed that the guard cells of *L. erecta* wild-type (WT) plants (the *pme6-1* background) showed strong binding of LM19 (Figures 2A and 2B), indicating an abundance of relatively unesterified pectin. There was an absence of LM20 binding (Figures 2E and 2F), indicating that highly methyl-esterified pectin is absent from the guard cells. In contrast, *pme6-1* plants had a reduction in the levels of de-esterified pectin in the guard cell, as indicated by the weaker binding of LM19 (Figures 2C and 2D) and abundant highly esterified pectin, as indicated by LM20 binding (Figures 2G and 2H). These data indicate that the structure of the HGA component of the pectin network has been altered in the *pme6-1* knockout line and, in particular, that the pectin of guard cells is more highly methyl-esterified in plants lacking *PME6*. JIM7-binding patterns remained consistent between the WT and *pme6-1* (Figures 2I–2L), indicating that the differences observed in Figures 2A–2H were due to an alteration in the methyl-esterification status of the guard cells rather than a change in the overall distribution of HGA in the cell wall. The pattern of methyl-esterification in guard cells was highly reproducible, as shown in Figures 2Q–2S. *pme6-1* lines complemented with a *proPME6*:*:PME6* construct showed a restoration of the WT methyl-esterification pattern (Figure S3). Analysis of controls (Figures 2M–2P) indicated that the patterns observed in Figures 2A–2L did not simply reflect patterns of cell wall thickness or overall distribution of cellulose. These data indicate that *PME6* is crucial for the de-methyl-esterification of guard cell wall HGA.

### A Mutant with Altered Guard Cell Wall Pectin Methylation Has Impaired Stomatal Function

**Figure 3:**
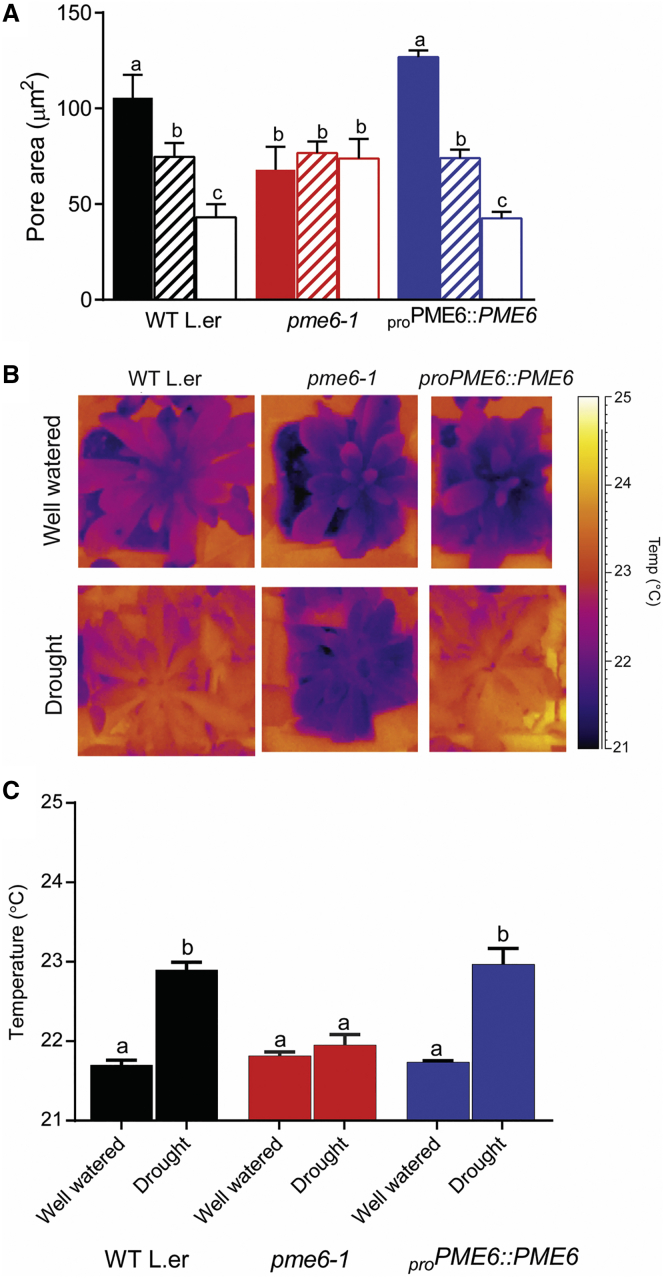
*pme6-1* Plants Have Altered Guard Cell Physiology and Water Relationships (A) Guard cell opening/closure response to changing CO_2_ concentration is lost in the *pme6-1* mutant. Pore area was measured from stomata in epidermal peels taken from the genotypes indicated (WT, *pme6-1*, and *pme6-1* complemented with a _pro_PME6::PME6 construct) after incubation of the peels with either CO_2_-free air (0 ppm CO_2_; solid bars), ambient CO_2_ (hatched bars) or high CO_2_ (1,000 ppm; open bars). Each column shows the mean and SEM. (n = 6), with statistical differences determined by ANOVA with a post hoc Tukey test. Columns indicated with identical letters cannot be distinguished from each other (p < 0.01). (B) *pme6-1* plants are less able to adjust leaf temperature under drought conditions. Thermal images are shown of well-watered plants of the genotypes indicated (top images) taken at day 0 post-drought. Images of equivalent plants at day 5 post-drought (lower panel) show that the *pme6-1* plants have a lower leaf temperature than the WT or the complemented *pme6-1* mutant. (C) Quantification of thermal image data shows that *pme6-1* leaf temperature does not change significantly under drought conditions, while the WT and the complemented mutant leaf temperature increases. Each bar represents the mean temperature for the rosette with error bars indicating SEM (n = 6). Statistical differences were determined by ANOVA with a post hoc Tukey test. Columns indicated with identical letters cannot be distinguished from each other (p < 0.05). See also Figure S4.

To study the functional significance of the changing pectin methyl-esterification status of the guard cells, we investigated the stomatal opening and closure responses of *pme6-1*. Figure 3A shows the stomatal aperture response in isolated epidermal strips exposed to buffers supplied with elevated (1,000 ppm) or decreased (0 ppm) levels of CO_2_. Exposure to elevated CO_2_ caused WT stomatal apertures to decrease, and CO_2_-free air caused WT apertures to increase, as previously reported [17]. In contrast, *pme6-1* stomatal apertures were relatively insensitive to CO_2,_ with the responses to both elevated and decreased CO_2_ being lost. The stomatal aperture response to CO_2_ was restored in the complemented lines. A restricted ability of *pme6-1* stomata to respond to abscisic acid, a classical regulator of stomatal function [17], was also observed (Figure S4E), suggesting that the altered pectin methyl-esterification status of the guard cells was affecting a fundamental property of the stomata.

**Figure 4:**
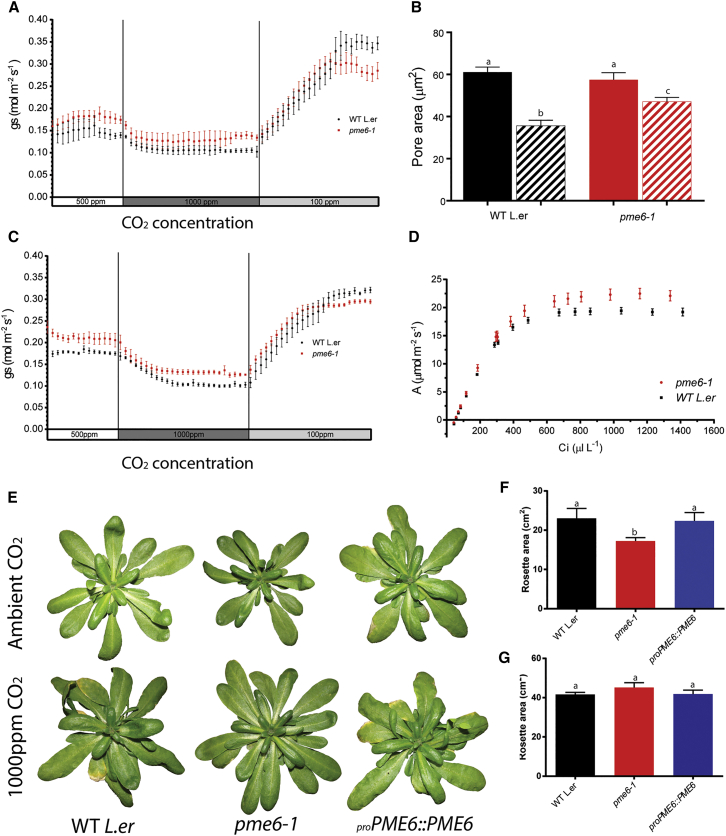
*pme6-1* Plants Show a Limited Dynamic Range of Stomatal Movement and Decreased Growth under Ambient CO_2_, which Is Rescued by Elevated CO_2_ (A) *pme6-1* leaves show a limited dynamic range in stomatal conductance (*g*_s_) in response to changing CO_2_ level. Gas exchange data for WT and *pme6-1* leaves show that, under ambient CO_2_ conditions, the *pme6-1* leaves have higher *g*_s_ than the WT. Following exposure to elevated (1,000 ppm) CO_2_, *g*_s_ in both mutants and WTs fall. Exposure to a low (100-ppm) CO_2_ regime induces increased *g*_s_, but the *pme6-1 g*_s_ trace plateaus to a lower value than for WT leaves. Error bars indicate the SEM (n = 8). (B) *pme6-1* stomata show a differential pore size response after incubation in high osmoticum. Stomatal pore areas were measured in epidermal peels from either WT or *pme6-1* leaves incubated either in resting buffer (solid bars) or resting buffer with addition of mannitol to 0.5 M (hatched bars). Statistical differences were determined by ANOVA and a post hoc Tukey test. Columns indicated with identical letters cannot be distinguished from each other (p < 0.01, n = 3, with 40 stomata counted from a total of four plants, repeated on 3 consecutive days). Error bars indicate the SEM. (C) The more limited dynamic range in *g*_s_ exhibited by *pme6-1* leaves is maintained after the growth of plants at elevated CO_2_. Gas exchange data for WT and *pme6-1* leaves taken from plants grown continually under elevated CO_2_. The traces for the WT and *pme6-1* as the CO_2_ level is altered during gas exchange analysis are comparable to those shown in (A), with the *pme6-1* trace again reaching a lower plateau after exposure to sub-ambient CO_2_ level (n = 8). (D) At elevated CO_2_ levels, the *pme6-1* leaves have a greater potential to assimilate CO_2_ than WT leaves. A/Ci curve analysis of WT and *pme6-1* leaves indicates that the instantaneous C assimilation rate at ambient CO_2_ levels is comparable but that as C_i_ increases, the *pme6-1* leaves show a greater maximum potential assimilation rate (n = 5 for WT; n = 6 for *pme6-1*; error bars indicate the SEM). (E–G) *pme6-1* plants are smaller than WTs under ambient CO_2_, but growth at elevated CO_2_ leads to plants attaining a similar size. Images of plants (genotypes as indicated) under ambient CO_2_ are shown in (E, top row) and under elevated (1,000 ppm) CO_2_ in (E, bottom row). Quantitation of total rosette area of plants grown under ambient CO_2_ (F) shows that *pme6-1* plants achieve a smaller final size, whereas growth in elevated CO_2_ (G) leads to all plants reaching a similar mean size. In (F and G), error bars indicate the SEM, n = 8. See also Figure S4.

Since stomata play a major role in controlling the water relations of the plant, thermal imaging was used to investigate the effects of the *pme6-1*-altered guard cell wall properties at the whole-plant level by gauging leaf temperature as a measure of evaporative cooling (which is tightly linked to stomatal function [18]). Under well-watered growth conditions, there were minimal differences in temperature between the *pme6-1*, the WT, and complemented mutant lines (Figure 3B). However, under drought conditions, *pme6-1* plants were significantly cooler than the WT and complemented lines (Figures 3B and 3C), probably due to a higher rate of transpiration through their more open stomata. These results are consistent with the data in Figure 3A, indicating that the *pme6-1* mutant stomata have a more restricted range of stomatal opening/closure as a result of altered guard cell wall properties and a more restricted response to ABA (Figure S4). To further investigate the physiological outcome of altered stomatal performance in the *pme6-1* mutant, we conducted infrared gas exchange analysis to assess stomatal conductance (*g*_s_), in response to shifts in CO_2_ conditions. Under ambient CO_2_ conditions, the *pme6-1* leaves had a higher *g*_s_ compared to the WT (Figure 4A). When exposed to elevated CO_2_, the *pme6-1 g*_s_ value decreased slightly but remained higher than the WT value. However, when the CO_2_ level was decreased to sub-ambient, both *pme6-1* and WT *g*_s_ increased but the maximal level achieved by the *pme6-1* leaves plateaued at a lower level than that of the WT; thus, the overall dynamic range of *g*_s_ shown by the *pme6-1* leaves was lower than for the WT. Unlike stomatal aperture measurements in epidermal peels, *g*_s_ is influenced not only by the guard cells but also by a variety of physiological processes in the whole leaf and plant, which may counteract the actions of individual stomata, leading to an amelioration of the response seen in isolated peels [19]. The data in Figure 4A indicate that *pme6-1* stomata have a more restricted dynamic range of opening/closure that cannot be completely compensated for at the whole plant level, thus leading to altered water relationships.

An altered dynamic range of guard cell swelling and deflation was also indicated by direct observation of stomatal pore area after immersion in an osmoticum expected to decrease turgor pressure [8]. Despite being subjected to a similar decrease in osmotic potential (1.23 MPa), the *pme6-1* stomatal pores remained significantly larger than those of the WT (Figure 4B). Analysis of guard cells by electron microscopy did not reveal any overt difference in surface shape or size (Figures S2E and S2F), and overall cell ultrastructure appeared similar in the two genotypes (Figures S2G and S2H), suggesting that the different behavior of the stomata was not due to large-scale change in cell structure but rather to some alteration in the mechanical properties of the cell wall. The differential response to a similarly imposed biophysical challenge via mannitol treatment supports the idea that the pectin structure in the guard cells sets the mechanics of the cellular complex, thus limiting the range of size change possible. It has been postulated that the methyl-esterification status of pectin influences the ability of HGA domains to associate via Ca^2+^ cross-links and that the degree of Ca^2+^ cross-linking has a major effect on the mechanical stiffness of the cell wall matrix. Indeed, previous work on guard cell walls suggested that arabinan side chains of the rhamnogalacturonan-I pectic domain associated with HGA might play a role in physically separating HGA domains, thus influencing Ca^2+^ cross-linking [8]. Since the formation of pectic network structures will be strongly dependent on the methyl-esterification status of HGA, PMEs can be predicted to play a major role in determining the overall mechanical properties of the cell wall. However, although recent evidence strongly supports the role of pectin methyl-esterification status in cell wall mechanics [20, 21, 22], simple inference of mechanics based on pectin methyl-esterification has proven problematic, since it appears to be highly dependent on cellular context [23, 24]. Our data suggest a situation in which a reduction in pectin de-esterification leads to guard cells that are relatively stiffer, limiting potential changes in cell size.

### The Influence of Altered Guard Cell Wall Structure on Plant Growth Is Environment Dependent

From our gas exchange analysis, we derived A/Ci (photosynthesis rate/internal CO_2_ concentration) curves relating instantaneous carbon assimilation rate to CO_2_ level (Figure 4D). At ambient CO_2_, both *pme6-1* and WT leaves showed similar assimilation rates; however, at elevated CO_2_, a much higher rate was measured in *pme6-1*. As CO_2_ level rises relative to O_2_, it is expected that photorespiration in C3 plants (such as *Arabidopsis*) will decrease [25], thus leading to a higher net assimilation rate. Leaves with stomata that show a decreased closure response to CO_2_, as observed here for *pme6-1*, might be expected to have higher internal CO_2_ levels and, thus, a greater increase in assimilation rate than the WT, as indicated in Figure 4D. To investigate the outcome of this at a whole-plant level, we compared the growth of WT and *pme6-1* mutant plants under ambient and elevated CO_2_. *pme6-1* plants were smaller than WTs when grown under ambient CO_2_ (Figures 4E and 4F). When grown under elevated CO_2_, the *pme6-1* and WT plants were larger than the equivalent plants grown under ambient CO_2_ (as expected) [26], but there was no difference in size between mutants and WTs (Figures 4E and 4G). Gas exchange analysis confirmed that the more limited dynamic range in *g*_s_ observed in the *pme6-1* leaves under ambient CO_2_ conditions was maintained when the plants were grown under elevated CO_2_ (Figure 4C), indicating that the underlying, more limited, dynamic range of stomatal function was also present in the mutant under these growth conditions, as expected for a genetically determined change in cell wall structure.

Stomatal size and density are well-characterized parameters known to influence leaf performance under various environments [26]. In the wake of rising atmospheric CO_2_ levels, there has been much interest in understanding how stomatal parameters provide an insight into past evolutionary events linked to earlier environments [27] and into the potential modification of stomata to create crop plants better attuned to present and predicted climates [28, 29]. The potential role of the guard cell wall in setting and modulating the response dynamics of stomata has been underexplored. Since our analysis did not indicate any differences in stomatal density or stomatal index between *pme6-1* and WT leaves (Figures S4A–S4D), the most plausible interpretation of our data is that that modulation of the pectin matrix of guard cells leads to altered wall properties so that the stomata are mechanically limited in their responses to exogenous cues, thus altering plant-water relations.

Despite a firm theoretical basis for the importance of differential guard cell wall stiffness in the mechanism of stomata opening and closure in response to altered turgor pressure [1, 30], experimental evidence has often been correlative, e.g., measurements of cell wall thickening, observations of cellulose microfibril orientation [2]. Indeed, despite a wealth of physiological data on turgor pressure and ion fluxes [31], and intricate details of molecular signaling in guard cells [17], the causal relationship between guard cell wall structure/composition and stomatal function has been surprisingly underexplored. The most insightful data have come from experiments in which the exogenous supply of cell-wall-modifying enzymes suggested an important role for pectic arabinans in stomatal function [8]. However, the nature of the genes involved and, indeed, formal genetic evidence to support this hypothesis, are lacking. In this paper, we provide molecular data to show that not only do *Arabidopsis* guard cells have a specific HGA methyl-esterification status (unesterified), but also that this status is required for normal stomatal function. The simplest interpretation of our data is that abnormal pectin methyl-esterification alters mechanical properties of the guard cells, leading to an inability to show appropriate opening and closure in response to environmental signals. Due to the importance of the mechanical properties of guard cells in setting the dynamics of stomatal opening and, thus, whole-plant/water relations, this alteration in guard-cell-specific cell wall gene expression leads to a poorer ability of the leaf to control water loss under drought conditions and poorer growth of the plants under ambient CO_2_ levels. Interestingly, this growth defect was overcome when the mutant plants were grown at elevated CO_2_, indicating that, under certain conditions, altered guard cell wall mechanics are not detrimental. In summary, our data indicate that, in addition to the well-explored regulation of stomatal dynamics via signal transduction signals acting on ion transport to vary turgor pressure, cell wall modification plays an important role in setting the overall limits of the system. Although this study focused on the role of pectins, it is clear that cell wall matrix components function together to influence mechanical properties [32], and exploring the roles of both structural carbohydrates, such as xyloglucans and cellulose [33], and modulating protein factors, such as expansins [34], will allow a deeper understanding of the system. Allied to this, targeted modulation of guard cell wall structure provides a novel avenue for the future manipulation of stomatal function.

## Experimental Procedures

Details of the experimental procedures are available in the Supplemental Information.

## Author Contributions

Conceptualization, A.J.F. and J.E.G.; Investigation, S.A., L.H., N.E., A.B., M.L., Y.V, and A.J.F.; Writing – Original Draft, A.J.F., J.E.G., and S.A.; Writing – Review & Editing, A.J.F., J.E.G., S.A., L.H., J.P.K., Y.V., and H.V.S.; Resources, J.P.K.; Supervision, A.J.F. and J.E.G.; Funding Acquisition, A.J.F., J.E.G., and H.V.S.

## Supplemental Information

Document S1. Supplemental Experimental Procedures, Figures S1–S4, and Table S1

Document S2. Article plus Supplemental Information
