# Supplementary material for: Stomatal Function Requires Pectin De-methyl-esterification of the Guard Cell Wall
Source: Curr Biol. 2016 Nov 7;26(21):2899–906. doi: 10.1016/j.cub.2016.08.021 (PMC5106435; doi:10.1016/j.cub.2016.08.021)
Supplement: Document S1. Supplemental Experimental Procedures, Figures S1–S4, and Table S1 [file mmc1.pdf]

Current Biology, Volume *26*

Supplemental Information

Stomatal Function Requires Pectin

De-methyl-esterification of the Guard Cell Wall

Sam Amsbury, Lee Hunt, Nagat Elhaddad, Alice Baillie, Marjorie Lundgren, Yves Verhertbruggen, Henrik V. Scheller, J. Paul Knox, Andrew J. Fleming, and Julie E. Gray

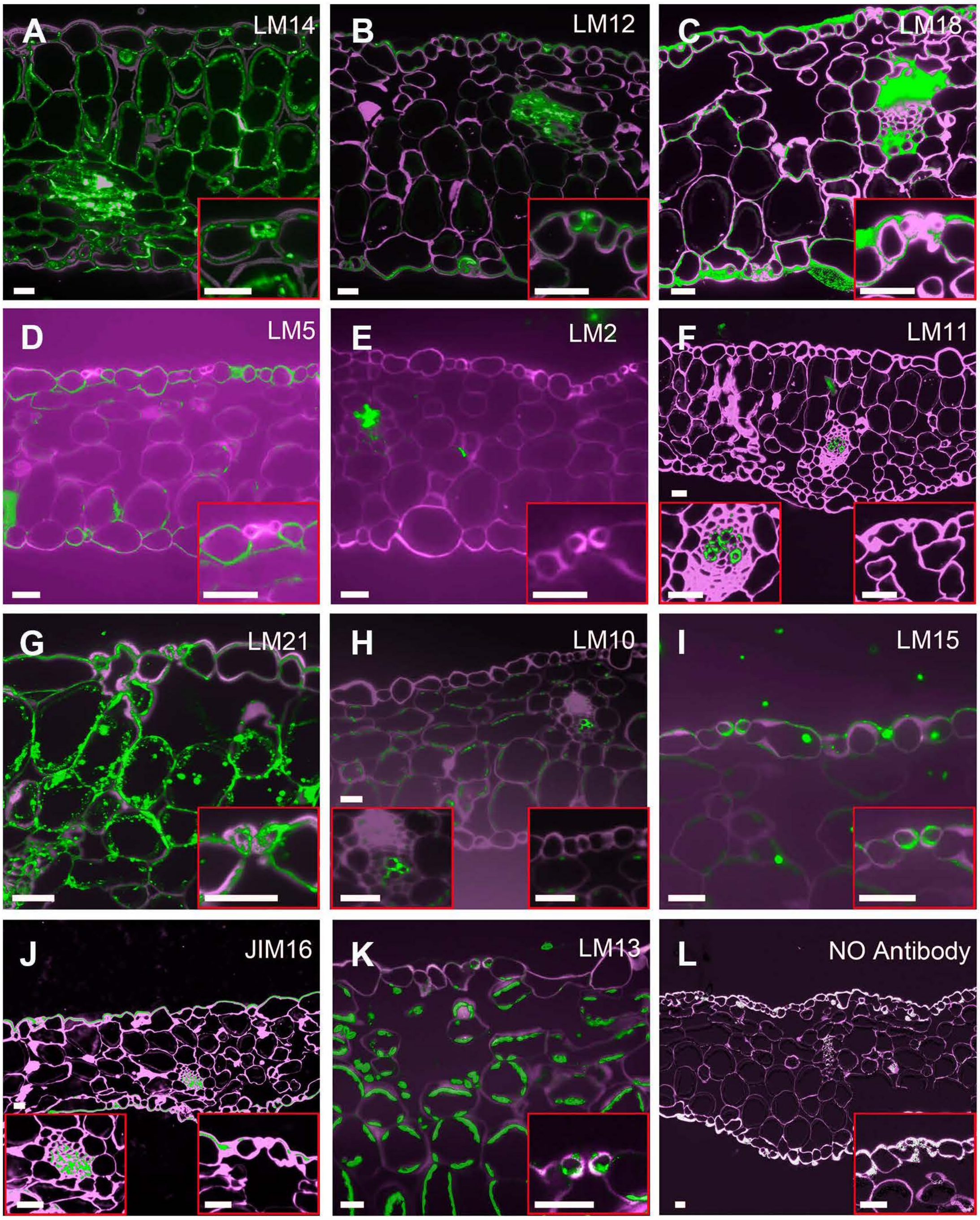

Figure S1. Related to Figure 1. A range of cell wall epitopes patterns are detected in the leaf by immunolabelling. (A) LM14 signal (green) indicates broad distribution of arabinogalactan protein in the mesophyll, epidermis and guard cells. (B) LM12 indicates feruloylated polymers are present in guard cells and vascular cells, with low levels of epidermal cell binding also present. (C) LM18 binding, which detects homogalacturonan which is partially methylesterified, was observed in the vasculature and the epidermis but not the guard cells. (D) LM5 binds (1-4)-β-D-Galactan and signal was detected in epidermal cells and sporadically in the mesophyll, with no guard cell binding observed. (E) LM2 binds an epitope of arabinogalactan proteins and was only detected in the vasculature. (F) LM11 binds xylan and arabinoxylan and was only detected in the vasculature. (G) LM21 recognises heteromannan. Strong signal was observed in the mesophyll with weaker but consistent binding in the epidermis and guard cells. (H) LM10 binds heteroxylan and was only detected in the vasculature. Signal in chloroplasts reflects autofluorescence (I) LM15 binds the XXXG motif of xyloglucan and binding was observed weakly in the mesophyll and epidermal cells and strongly in the guard cells. (J) JIM16 binds AGPs and was observed in the mesophyll and weakly in the epidermis but not in the guard cells. (K) LM13 binds arabinan and signal was observed in the guard cells. Signal in the chloroplasts reflects autofluorescence (L) Samples with no primary antibody show a low level of fluorescence in the green channel. Insets in A-L show detail either of guard cells (lower right in respective panel) or vasculature (lower left in respective panel). In all panels the green signal shows antibody signal following binding of the specific primary antibody indicated and the magenta signal (false-colour) indicates Calcofluor White fluorescence of cell walls.Scale bars = 20 μm.

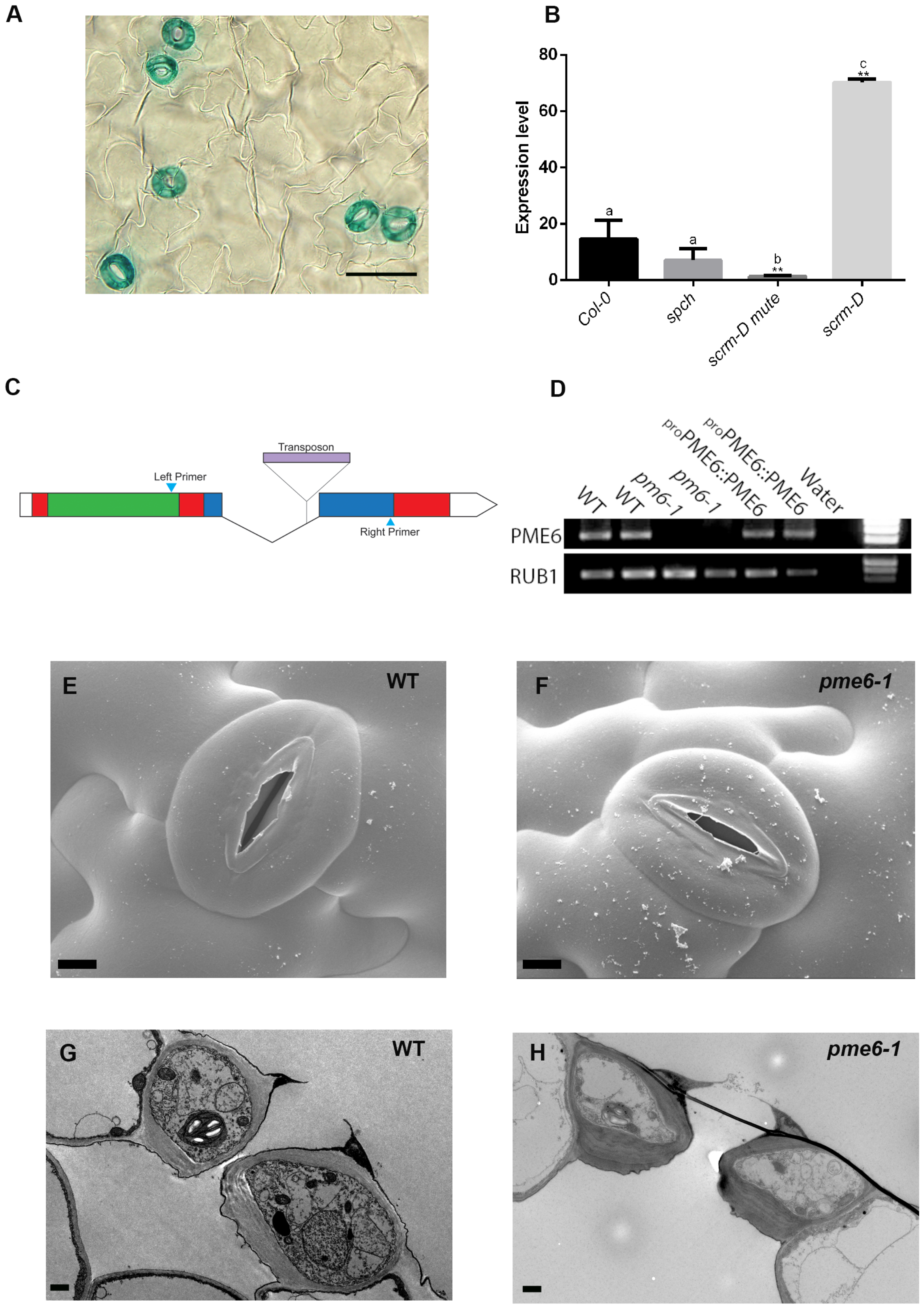
A
B
Expression level
80
60
40
20
0
a
a
b
**
c
**
Col-0
spch
scrm-D mute
scrm-D
C
Transposon
Left Primer
Right Primer
D
WT
WT
pm6-1
pm6-1
proPME6::PME6
proPME6::PME6
Water
PME6
RUB1
E
WT
F
pme6-1
G
WT
H
pme6-1

Figure S2

Figure S2. Related to Figure 2. *PME6* expression is up-regulated in guard cells but loss of *PME6* does not lead to a major change in guard cell shape and structure. (A) Histochemical staining of Arabidopsis leaves containing a $_{pro}$PME::GUS construct. Signal (blue) is observed only in mature guard cells. (B) *PME6* transcript levels are relatively high in the *scrm-D* mutant which has an increased number of mature stomata whereas *PME6* transcript levels are relatively low in mutants with an increased number of pavement cells (*spch*) or meristemoids (*scrm-D mute*). Columns indicate mean values, error bars = s.e.m. (n=5). Expression levels indicated with the same letter cannot be distinguished from each other at the 0.05 confidence limit (ANOVA and post-hoc Tukey, n= XX). (C) Schematic of the *pme6-1* locus containing a DS insertion mutant. The insertion is in an intron (blue) within the 3'UTR (red). Left and right primer sites used for PCR are indicated. (D) RT-PCR analysis of *PME6* transcript level in WT (*Landsberg erecta*), *pme6-1* mutant, and complemented *pme6-1* mutant lines. No transcript was detected in the *pme6-1* mutant line. Transcripts were detected in all samples using primers for the control gene *RUB1*. (E) cryoSEM image of a wild-type stomate and (F) a *pme6-1* stomate. (G) TEM of a wild-type stomata and (H) a *pme6-1* stomate. Scale bars = 25 μm in A; 4μm in E,F; 2 μm in G,H.

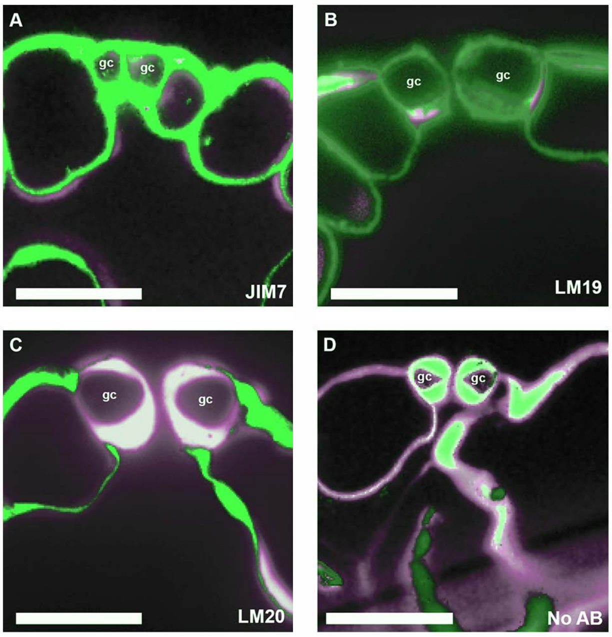

Figure S3. Related to Figure 2. Pectin methylation pattern in guard cells is restored to WT in complemented *pme6-1* plants. (A) JIM7 antibody labelling of the epidermis of the *pme6-1* mutant complemented with the $_{pro}$PME6::PME6 construct reveals that HGA is present in all cell walls. (B) Relatively un-esterified HGA, revealed using the LM19 antibody, is present in all cell walls of the complemented mutant, including those of the guard cells (C) Methyl esterified HGA, revealed using the LM20 antibody, is excluded from the guard cell wall in a pattern similar to that observed in WT plants (compare Fig 2E). (D) Controls with no primary antibody reveal a low level of autofluorescence. Antibody binding is indicated by green signal whereas magenta signal indicates Calcofluor staining of the cell wall. Observed patterns were consistent when replicated (n=8). Guard cells indicated by gc. Scale bars represent 20 μm.

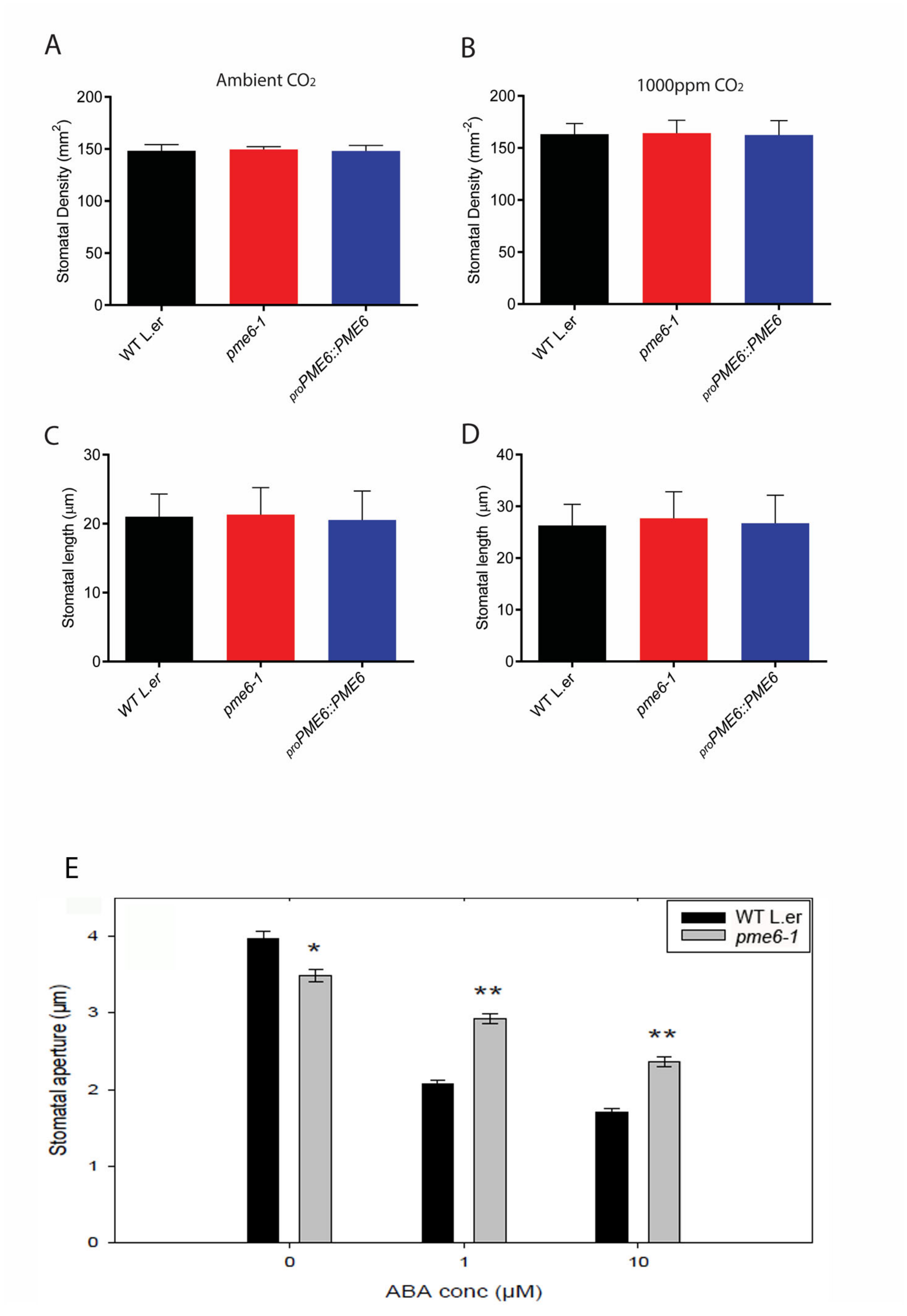

Figure S4. Related to Figures 3 and 4. *pme6-1* stomata are less responsive to ABA induced closure but stomatal size and density are unchanged (A) Stomatal density in leaves from WT, *pme6-1* and *pme6-1* complemented plants grown under ambient CO2 or (B) elevated (1000 ppm) CO2. (C) Stomatal length in leaves from WT, *pme6-1* and *pme6-1* complemented plants grown under ambient CO2 or (D) elevated (1000 ppm) CO2. Columns indicate mean values, error bars = s.e.m. (n=5). (E) Epidermal peels were taken from WT or *pme6-1* leaves and incubated for 2 hours in the light (300µmol m-2 s-1) in opening buffer supplied with CO2 free air before addition of ABA to 1 or 10µM, as indicated. Apertures were measured after incubation for a further 2 hours. Each column indicates the mean stomatal aperture achieved with error bars indicating s.e.m. A t-test was performed on each pair of measurements (WT vs *pme6-1*) with single asterisk (*) indicating a significant difference at p = 0.05 and double asterisk (**) indicating a significant difference at p = 0.01.

Table S1 Summary of primary antibodies used and the binding pattern observed.

Antibody	Cell wall epitope	Binding in mature leaves	References
	**Hemicelluloses**		
LM10	(1-4)-B-D-xylan	Yes: Vasculature	[S5]
LM11	(1-4)-B-D-xylan/arabinoxylan	Yes: Vasculature	
LM15	Xyloglucan (XXXG)	Yes: Guard cells and weakly in epidermal cells	[S6]
LM24	Xyloglucan	None	[S7]
LM25	Xyloglucan	None	
LM21	Mannan	Yes: Broad binding	[S8]
LM22	Mannan	None	
	**Pectins**		
JIM5	Partially methylated homogalacturonan	None	[S9]
JIM7	Partially/completely de-esterified homogalacturonan	Yes: Broad binding	
2F4	Calcium-crosslinked pectins	Yes: Guard cell junctions	[S10]
Pam1	Blockwise de-esterified homogalacturonan	None	[S11]
LM7	Non-blockwise de-esterified homogalacturonan	None	[S12]
LM18	Partially de-esterified HGA	Yes: Broad binding. Less binding in guard cells	[S9]
LM19	unesterified homogalacturonan	Yes: Broad binding	
LM20	highly esterified homogalacturonan	Yes: Yes, absent from guard cells	
LM8	Xylogalacturonan	None	[S13]
LM5	(1->4)-b-D-galactan	Yes: Epidermis, absent from guard cells	[S14]
LM9	Feryloylated (1->4)-b-D-galactan	None	[S15]
LM6	(1->5)-a-L-arabinan	None	[S16]
LM13	Linearised-(1->5)-a-L-arabinan	Yes: Weak binding, present in guard cell ledges	[S17]
LM16	Processed arabinan	None	[S18]
	**Other glycans**		
LM12	Feruloylated arabinosyl/galactosyl	Yes: Epidermal, guard cell and vasculature binding	[S7]
LM23	Xylogalacturonan	None	

	**Extensins**		
LM1	Extensin	None	[S19]
JIM11	Extensin	None	[S20]
JIM12	Extensin	None	
JIM19	Extensin	None	[S19]
JIM20	Extensin	None	[S20]
	AGP'S		
LM2	b-linked GlcA	Yes: Vasculature	[S20]
LM14	AGP glycan	Yes: Broad binding	[S17]
JIM4	AGP glycan	None	[S21]
JIM8	AGP glycan	None	[S22]
JIM13	AGP glycan	None	[S23]
JIM14	AGP glycan	None	
JIM15	AGP glycan	None	
JIM16	AGP glycan	Yes: Epidermal and vasculature, less binding in guard cells	
MAC207	AGP glycan	None	

Supplemental Experimental Procedures

Plant material

Seeds were surface sterilised in bleach diluted in water (1:5 v/v) containing 0.05% (v/v) Tween20 and stratified at 4°C for 7 days. Seeds were then transferred to square pots of 6 cm diameter and 8 cm height containing 1:3 mix of perlite:soil and transferred to a controlled environment chamber and grown with 12 h light (200 µmol m^{-2} s^{-1}) with 22°C day temperature, 16°C night temperature and 60% humidity. Plants used for immunolabelling were taken at 21 days, and, plants used for aperture analysis and gas exchange at 28 days after transfer to the growth chamber.

For creation of the *PME6* promoter GUS reporter line, a region approximately 1200 bp upstream of the ATG promoter translational start codon of *PME6* was amplified from genomic DNA with primers 5′-CACCTGGGATCCAAAATGATTG-3'5'- TGTGGGATATTGTTTTCTTAGGG -3′ and KOD DNA polymerase, inserted into the pENTR-D-TOPO entry vector (Invitrogen), and recombined with the pKGWFS7 destination vector [S1] before transfer into *Agrobacterium tumefaciensC58* cells and transformation into Col-0 Arabidopsis by floral dip [S2]. Seeds were selected on 50 µgml^{-1} kanamycin and insertion confirmed by PCR using forward $_{Pro}$PME6 specific primer and GUS gene reverse primer (5′-TGCTCAGGTAGTGGTTGTCG-3′).

The *pme-6* T-DNA insertion line (SGT6342) was obtained from NASC (Nottingham,UK) and confirmed as homozygous for the insertion by PCR using primers 5′-TCTGAGTCGTGTAAACGAGCC - and 5′-CCTCTTCGTATTCAAAGTATTTCCC. To create the *pme6-1* line complemented with *PME6* the coding region of *PME6* was amplified from the vector pUNi-At1g23200 (U18916;ABRC) with primers 5'-CACCAACCTAAACAAAAAAACC- and 5'-GATGACAACCGATTAAATTAATAAC and recombined into the pENTR-D-TOPO vector. This was then recombined by LR reaction into pMDC32 [S3] then excised with AscI. The pENTR-D-TOPO containing the *PME6* promoter (described above) was cut with AscI and the *PME6* coding region ligated 3' of the promoter. The plasmid was recombined by an LR reaction with pHGW [S1] to create the proPME6::PME6 construct before transfer into *Agrobacterium tumefaciensC58* cells and transformation into the *pme6-1* background by floral dip [S2]. The complemented *pme6-1* line is referred to as $_{pro}$PME6::PME6. Transformants were selected on 0.5X MS (Murashige and Skoog) medium, 1.5% (w/v) sucrose containing 15mgL^{-1} hygromycin and plants from the T3 generation analysed.

Gene expression analysis and immunolabelling

For analysis of *PME6* expression, RNA was extracted from seedlings using a Qiagen RNAeasy kit, and reverse transcribed into cDNA using oligo dT primer and SuperscriptII (Invitrogen). PCR was carried out on cDNA to determine if any transcript was detectable. Primer 5'-GGAAGATTCCAAAACTACGGC and 5'-GCCGTCCTAAATAAGTTTCCG were used to detect *PME-6* transcript, RUB1 (AT4G36800) primers were used as a positive control; (5'-GCGAACTTCGTCTTCACAA and 5'-GGAAAAAGGTCTGACCGACA.

Histochemical staining for GUS activity was carried out on leaves of T2 seedlings in 50 mM potassium phosphate, 1 mM potassium ferrocyanide, 1 mM potassium ferricyanide, 0.2% (v/v) Triton X-100, 2 mM 5-bromo-4-chloro-3-indolyl-β-d-glucuronic acid, and 10 mM EDTA after vacuum infiltration at 37°C. Leaves were decolorized overnight with 70% (v/v) ethanol, and washed in 10% glycerol. Images were captured with an Olympus BX51 microscope connected to a DP70 digital camera. Expression patterns shown were typical of several independently transformed lines.

For immunolabelling, 21-day-old leaf samples (3 mm squares) were fixed in 4% (w/v) formaldehyde in PEM buffer (0.1 M PIPES, 2 mM EGTA, 1 mM $MgSO_4$, adjusted to pH 7) by vacuum infiltration then dehydrated in an ethanol series (30 min each at 30%, 50%, 70%, 100%

EtOH) and infiltrated with LR White Resin (London Resin Company) diluted in ethanol (45 min each at 10%, 20%, 30%, 50%, 70% & 90% resin then 3x8 h at 100%). Samples were stood vertically in gelatine capsules filled with resin and allowed to polymerise for 7 days at 37°C. Sections were cut to a thickness of 2 µm using a Reichert-Jung Ultracut E ultramicrotome using a glass knife.

Sections were incubated with 3% (w/v) milk protein (Marvel, Premier Beverages, UK) in phosphate-buffered saline solution (PBS, pH 7.2) (hereafter known as PBS/MP). Sections were then incubated with a ten-fold dilution of primary monoclonal antibody in PBS/MP for 1 h at room temperature. Samples were washed 3 times with PBS and secondary antibody was added (100-fold dilution in PBS/MP) for 1 h. Samples were kept in the dark from this step. For the JIM- and LM- series of antibodies anti-rat-IgG (whole molecule) coupled to fluorescein isothiocyanate (FITC) was used, for the 2F4 antibody, an anti-mouse-IgG (whole molecule) coupled to FITC was used. Samples were counterstained with 0.25% (w/v) Calcofluor White solution diluted ten-fold in PBS for 5 min before mounting on slides with Citifluor AF1 anti-fade solution (Agar Scientific, UK). Samples were visualised on an Olympus BX51 microscope with epifluorescence optics fitted and images captured using a DP51 camera. FITC was visualised using a filter set with 460-490 nm excitation filter, a 510-550 nm emission filter and a 505 nm dichroic mirror. Calcofluor White was visualised using a 400-410 nm excitation filter, a 455 nm emission filter and a 455 nm dichroic mirror.

The reproducibility of antibody patterns were assessed by a scoring technique. 50 stomata were assessed and the pattern of immunolabelling was classed in terms of its prevalence in the guard cells. Guard cells which were fully labelled with antibody, as typified by JIM7 labelling were classed as “Fully” labelled, guard cells which had some signal in the guard cell but not distributed throughout the whole cell were classed as “partial” and stomata which had no labelling inside the guard cell but did show signal at the junctions between guard cells and their neighbouring cells were classed as “Junctions only”. No stomata analysed fell outside of these three categories.

Electron Microscopy

For cryo-scanning electron microscopy (cryo-SEM), leaves were careful removed with forceps and placed flat on a brass stub, stuck down with a cryo glue preparation consisting of a 3:1 mixure of Tissue-Tec (Scigen Scientific, USA)) and Aquadag colloidal graphite (Agar Scientific, Stansted, UK)) and then plunge frozen in liquid nitrogen with vacuum applied. For sample preparation for cryo fracture, leaves were placed vertically in recessed stubs held by the cryo glue preparation. Frozen samples were then transferred under vacuum to the prep chamber of a PT3010T cryo-apparatus (Quorum Technologies, Lewes, UK) and maintained at -145C. Surface ice was removed using a sublimation protocol consisting of -90C for 3 min. For cryofracture, no sublimation was carried out and instead a level semi-rotary cryo knife was used to randomly fracture the leaf. All samples were sputter coated with platinum until a measured thickness of 5 nm was recorded. Samples werethen transferred and maintained cold, under vacuum into the chamber of a Zeiss EVO HD15 SEM fitted with a cryo-stage. Images were taken on the SEM using a gun voltage of 6 kV, I probe size of 460 pA, a SE detector and a working distance of between 5 and 6mm. For transmission electron microscopy, leaves were dissected into 3% (w/v) glutaraldehyde (Sigma-Aldrich) in 0.1 M phosphate buffer. Further fixation and processing were as described previously [S4]

Stomatal aperture measurements

Abaxial epidermal peels of mature leaves were removed at least 2 hours into the photoperiod and floated onto opening buffer (10 mM KCl, 10 mM MES, pH 6.2). Samples were maintained at 22°C with 200 µmol $m^{-2} s^{-1}$ of light. For CO_2 responses air was bubbled into the opening buffer containing either 0 ppm CO_2 (CO_2 free treatment), ambient CO_2, or 1000ppm CO_2. For mannitol response

samples, 0.5 M mannitol was bubbled into the opening buffer. For ABA responses epidermal peels were incubated in opening buffer supplied with CO_2 free air for two hours before ABA was added to the buffer. For mannitol responses peels were incubated in 0.5M mannitol added to buffer (10 mM MES, pH6.2). Epidermal peels were imaged after 2 hours using an Olympus BX51 microscope and DP70 digital camera and stomatal apertures measured. 40 stomatal apertures were measured for each treatment in each of three independent experiments. For each experiment epidermal peels were taken from at least 3 plants of each genotype.

Thermal imaging

Infrared images were taken using a FLIR SC660 camera (FLIR systems). The camera was positioned 1 m above the leaf rosette. Plants were imaged at 24-days old under well-watered conditions at which point water was withheld. Plants were then imaged again at 29 days under strong drought conditions. 6 plants of each genotype were imaged and subsequent analysis was conducted using ThermaCAM researcher v2.10 professional (FLIR systems).

Gas exchange analysis

CO_2 shifts were conducted on 28-day old plants using mature non-senescent leaves. Analysis was started 2 hours into the photoperiod of the growth chamber and did not continue into the last 3 hours of the photoperiod. Measurements were taken using a LI-6400 infrared gas exchange analyser system using a leaf fluorometer chamber (LI-COR Inc.) with a $2cm^2$ circular area for measurement. Temperature was held at 21°C and humidity was kept above 58% and below 65%. Photon flux density was held at 300 µmol m^{-2} s^{-1} with 10% blue light. In cases where the leaf did not fill the chamber, leaf area was measured and a correction made in subsequent analysis. To assess stomatal response to CO_2 conductance was stabilised at 500 ppm for 40 minutes, CO_2 was then shifted to 1000 ppm for 50 minutes to stimulate stomatal closing, and then to 100ppm for 50 minutes to stimulate stomatal closure. A/Ci response curves were measured on young fully expanded leaves at 21°C leaf temperature, 1200 µmol m^{-2} s^{-1} PPFD light, and approximately 60% relative humidity. Once leaves were acclimated to chamber conditions, measurements were taken at 400, 250, 150, 100, 80, 60, and 40 every 2-3 minutes at 200 µmol s^{-1} flow rate, then at 400, 500, 600, 800, 900, 1000, 1200, 1400, and 1600 every 3-5 minutes at 300 µmol s^{-1} flow rate.

Analysis of stomatal size and density

For stomatal density analysis fully expanded non-senescent leaves were harvested from 35 day old seedlings. Leaves were fixed in 4% (v/v) formaldehyde in PEM buffer (0.1 M PIPES, 2 mM EGTA, 1 mM $MgSO_4$, adjusted to pH 7) for 8 hours. Leaves were then washed twice in 70% (v/v) ethanol for 30 minutes each wash. Tissue was then cleared by incubation in chloral hydrate (2.5 g mL-1) in 30% (v/v) glycerol twice for 8 h. Samples were then mounted in 30% (v/v) glycerol solution and imaged on an Olympus BX51 microscope under the 40x objective using Nomarsky illumination, images were captured with an Olympus DP70 camera and the number of stomata counted. 4 viewpoints per leaf were analysed and 3 leaves per plant.

For stomatal size analysis abaxial epidermal peels were taken and floated onto opening buffer ((10 mM KCl, 10 mM MES, pH 6.2). Samples were maintained at 22°C with 200µmol of light. CO_2 free air was bubbled through the buffer to promote stomatal opening. Epidermal peels were imaged after 2 hours using an Olympus BX51 microscope with a DP70 digital camera and stomatal complex length was measured.

Analysis of rosette area

Mature Arabidopsis plants were photographed at 30 days old from a height of 30cm using and Olympus E-PL1 digital camera. Rosette area was measured in ImageJ using the colour threshold tool to isolate the rosette in the image.

Supplemental references

S1. Karimi, M., Depicker, A., and Hilson, P. (2007). Recombinational cloning with plant gateway vectors. Plant Physiol. *145*, 1144–1154.

S2. Clough, S. J., and Bent, A. F. (1998). Floral dip: A simplified method for Agrobacterium-mediated transformation of Arabidopsis thaliana. Plant J. *16*, 735–743.

S3. Brand, L., Hörler, M., Nüesch, E., Vassalli, S., Barrell, P., Yang, W., Jefferson, R. a, Grossniklaus, U., and Curtis, M. D. (2006). A versatile and reliable two-component system for tissue-specific gene induction in Arabidopsis. Plant Physiol. *141*, 1194–1204.

S4. Wallace, S., Chater, C. C., Kamisugi, Y., Cuming, A. C., Wellman, C. H., Beerling, D. J., and Fleming, A. J. (2015). Conservation of Male Sterility 2 function during spore and pollen wall development supports an evolutionarily early recruitment of a core component in the sporopollenin biosynthetic pathway. New Phytol. *205*, 390–401.

S5. McCartney, L., Marcus, S. E., and Knox, J. P. (2005). Monoclonal Antibodies to Plant Cell Wall Xylans and Arabinoxylans\r10.1369/jhc.4B6578.2005. J. Histochem. Cytochem. *53*, 543–546.

S6. Marcus, S. E., Verhertbruggen, Y., Hervé, C., Ordaz-Ortiz, J. J., Farkas, V., Pedersen, H. L., Willats, W. G. T., and Knox, J. P. (2008). Pectic homogalacturonan masks abundant sets of xyloglucan epitopes in plant cell walls. BMC Plant Biol. *8*, 60.

S7. Pedersen, H. L., Fangel, J. U., McCleary, B., Ruzanski, C., Rydahl, M. G., Ralet, M. C., Farkas, V., Von Schantz, L., Marcus, S. E., Andersen, M. C. F., et al. (2012). Versatile high resolution oligosaccharide microarrays for plant glycobiology and cell wall research. J. Biol. Chem. *287*, 39429–39438.

S8. Marcus, S. E., Blake, A. W., Benians, T. A. S., Lee, K. J. D., Poyser, C., Donaldson, L., Leroux, O., Rogowski, A., Petersen, H. L., Boraston, A., et al. (2010). Restricted access of proteins to mannan polysaccharides in intact plant cell walls. Plant J. *64*, 191–203.

S9. Verhertbruggen, Y., Marcus, S. E., Haeger, A., Ordaz-Ortiz, J. J., and Knox, J. P. (2009). An extended set of monoclonal antibodies to pectic homogalacturonan. Carbohydr. Res. *344*, 1858–62.

S10. Liners, F., and Van Cutsem, P. (1992). Distribution of pectic polysaccharides throughout walls of suspension-cultured carrot cells - An immunocytochemical study. Protoplasma *170*, 10–21.

S11. Willats, W. G. T., Limberg, G., Buchholt, H. C., Van Alebeek, G. J., Benen, J., Christensen, T. M. I. E., Visser, J., Voragen, A., Mikkelsen, J. D., and Knox, J. P. (2000). Analysis of pectic epitopes recognised by hybridoma and phage display monoclonal antibodies using defined oligosaccharides, polysaccharides, and enzymatic degradation. Carbohydr. Res. *327*, 309–320.

S12. Willats, W. G. T., Orfila, C., Limberg, G., Buchholt, H. C., Van Alebeek, G. J. W. M., Voragen, A. G. J., Marcus, S. E., Christensen, T. M. I. E., Mikkelsen, J. D., Murray, B. S., et al. (2001). Modulation of the degree and pattern of methyl-esterification of pectic homogalacturonan in plant cell walls: Implications for pectin methyl esterase action, matrix properties, and cell adhesion. J. Biol. Chem. *276*, 19404–19413.

S13. Willats, W. G. T., McCartney, L., Steele-King, C. G., Marcus, S. E., Mort, A., Huisman, M., Van Alebeek, G. J., Schols, H. A., Voragen, A. G. J., Le Goff, A., et al. (2004). A xylogalacturonan epitope is specifically associated with plant cell detachment. Planta *218*, 673–681.

S14. Jones, L., Seymour, G. B., and Knox, J. P. (1997). Localization of Pectic Galactan in Tomato Cell Walls Using a Monoclonal Antibody Specific to (1[-]4)-[beta]-D-Galactan. Plant Physiol. *113*, 1405–1412.

S15. Clausen, M. H., Ralet, M.-C., Willats, W. G. T., McCartney, L., Marcus, S. E., Thibault, J.-F., and Knox, J. P. (2004). A monoclonal antibody to feruloylated-(1-->4)-beta-D-galactan. Planta *219*, 1036–41.

S16. Willats, W. G. T., Marcus, S. E., and Knox, J. P. (1998). Generation of a monoclonal antibody specific to (1→5)-α-l-arabinan. Carbohydr. Res. *308*, 149–152.

S17. Moller, I., Marcus, S. E., Haeger, A., Verhertbruggen, Y., Verhoef, R., Schols, H., Ulvskov, P., Mikkelsen, J. D., Knox, J. P., and Willats, W. (2008). High-throughput screening of monoclonal antibodies against plant cell wall glycans by hierarchical clustering of their carbohydrate microarray binding profiles. Glycoconj. J. *25*, 37–48.

S18. Verhertbruggen, Y., Marcus, S. E., Haeger, A., Verhoef, R., Schols, H. A., McCleary, B. V., McKee, L., Gilbert, H. J., and Knox, J. P. (2009). Developmental complexity of arabinan polysaccharides and their processing in plant cell walls. Plant J. *59*, 413–425.

S19. Smallwood, M., Martin, H., and Knox, J. P. (1995). An epitope of rice threonine- and hydroxyproline-rich glycoprotein is common to cell wall and hydrophobic plasma-membrane glycoproteins. Planta An Int. J. Plant Biol. *196*, 510–522.

S20. Smallwood, M., Beven, A., Donovan, N., Neill, S. J., Peart, J., Roberts, K., and Knox, J. P. (1994). Localization of cell wall proteins in relation to the developmental anatomy of the carrot root apex. Plant J. *5*, 237–246.

S21. Knox, J. P., Day, S., and Roberts, K. (1989). A set of cell surface glycoproteins forms an early marker of cell position , but not cell type , in the root apical meristem of Daucus carota L . Development *56*, 47–56.

S22. Pennell, R., Janniche, L., Kjellbom, P., Scofield, G., Peart, J., and Roberts, K. (1991). Developmental Regulation of a Plasma Membrane Arabinogalactan Protein Epitope in Oilseed Rape Flowers. Plant Cell *3*, 1317–1326.

S23. Yates, E. A., and Knox, J. P. (1994). Investigations into the occurrence of plant cell surface epitopes in exudate gums. Carbohydr. Polym. *24*, 281–286.
