# Supplementary material for: Stomatal Function Requires Pectin De-methyl-esterification of the Guard Cell Wall
Source: Curr Biol. 2016 Nov 7;26(21):2899–906. doi: 10.1016/j.cub.2016.08.021 (PMC5106435; doi:10.1016/j.cub.2016.08.021)
Supplement: Document S2. Article plus Supplemental Information [file mmc2.pdf]

Current Biology

Report

Stomatal Function Requires Pectin De-methyl-esterification of the Guard Cell Wall

Graphical Abstract

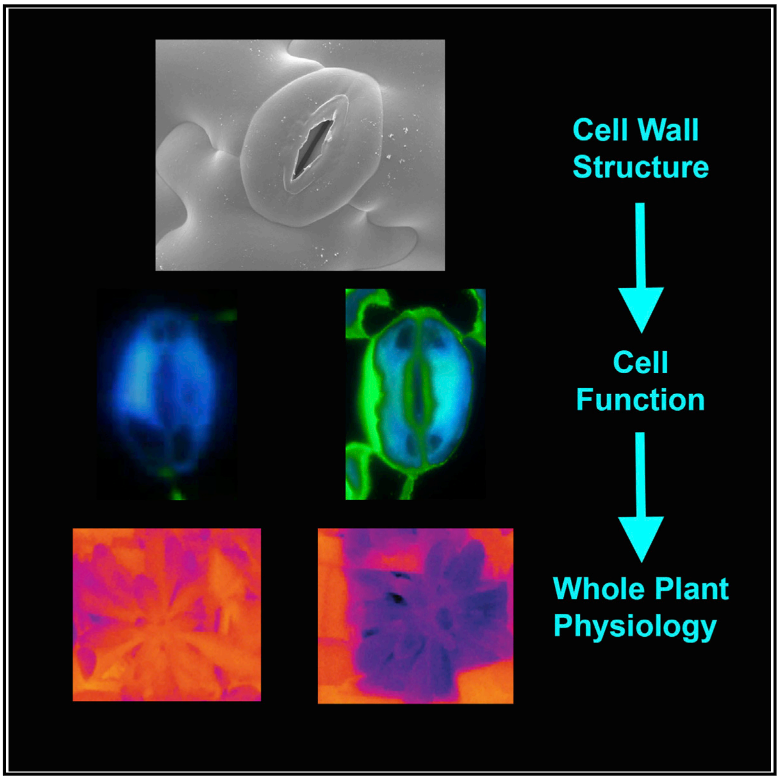

Highlights

- The guard cell wall is distinguished by a relatively low level of methylated pectin
- Increased methyl pectin leads to stomata with a smaller dynamic range of movement
- These plants show increased evaporative cooling and decreased growth under drought
- Elevated CO_2 restores mutant plant growth to normal

Authors

Sam Amsbury, Lee Hunt,
Nagat Elhaddad, ..., J. Paul Knox,
Andrew J. Fleming, Julie E. Gray

Correspondence

a.fleming@sheffield.ac.uk (A.J.F.),
j.e.gray@sheffield.ac.uk (J.E.G.)

In Brief

Guard cell wall mechanics must play a role in setting the dynamics of stomatal movement. Amsbury et al. show that the degree of pectin methylation in the wall sets the range of cell swelling, with consequences for plant water use and growth being dependent on CO_2 level. Stomatal mechanics are likely to influence plant response to climate change.

Amsbury et al., 2016, Current Biology *26*, 2899–2906
November 7, 2016 © 2016 The Authors. Published by Elsevier Ltd.
http://dx.doi.org/10.1016/j.cub.2016.08.021

Current Biology
Report

CellPress

Stomatal Function Requires Pectin De-methyl-esterification of the Guard Cell Wall

Sam Amsbury,[1] Lee Hunt,[2] Nagat Elhaddad,[2,3] Alice Baillie,[1] Marjorie Lundgren,[1] Yves Verhertbruggen,[4] Henrik V. Scheller,[4] J. Paul Knox,[5] Andrew J. Fleming,[1,6,*] and Julie E. Gray[2,*]
[1]Department of Animal and Plant Sciences, University of Sheffield, Sheffield S10 2TN, UK
[2]Department of Molecular Biology and Biotechnology, University of Sheffield, Sheffield S10 2TN, UK
[3]Department of Botany, University of Omar Al-Mukhtar, Al-Baida, Libya
[4]Biological Systems and Engineering Division and Joint BioEnergy Institute, Lawrence Berkeley National Laboratory, Berkeley, CA 94720, USA
[5]Centre for Plant Sciences, Faculty of Biological Sciences, University of Leeds, Leeds LS2 9JT, UK
[6]Lead Contact
*Correspondence: a.fleming@sheffield.ac.uk (A.J.F.), j.e.gray@sheffield.ac.uk (J.E.G.)
http://dx.doi.org/10.1016/j.cub.2016.08.021

SUMMARY

Stomatal opening and closure depends on changes in turgor pressure acting within guard cells to alter cell shape [1]. The extent of these shape changes is limited by the mechanical properties of the cells, which will be largely dependent on the structure of the cell walls. Although it has long been observed that guard cells are anisotropic due to differential thickening and the orientation of cellulose microfibrils [2], our understanding of the composition of the cell wall that allows them to undergo repeated swelling and deflation remains surprisingly poor. Here, we show that the walls of guard cells are rich in un-esterified pectins. We identify a pectin methylesterase gene, *PME6*, which is highly expressed in guard cells and required for stomatal function. *pme6-1* mutant guard cells have walls enriched in methyl-esterified pectin and show a decreased dynamic range in response to triggers of stomatal opening/closure, including elevated osmoticum, suggesting that abrogation of stomatal function reflects a mechanical change in the guard cell wall. Altered stomatal function leads to increased conductance and evaporative cooling, as well as decreased plant growth. The growth defect of the *pme6-1* mutant is rescued by maintaining the plants in elevated CO_2, substantiating gas exchange analyses, indicating that the mutant stomata can bestow an improved assimilation rate. Restoration of *PME6* rescues guard cell wall pectin methyl-esterification status, stomatal function, and plant growth. Our results establish a link between gene expression in guard cells and their cell wall properties, with a corresponding effect on stomatal function and plant physiology.

RESULTS AND DISCUSSION

Analysis of Guard Cell Wall Composition by an Antibody Screen

Probing *Arabidopsis thaliana* leaf sections with a panel of 36 monoclonal antibodies by fluorescence microscopy revealed a range of antibody-binding patterns, including clear differences in the composition of guard cell walls compared to epidermal or mesophyll cells (Figure 1; Figure S1; Table S1). Homogalacturonan (HGA) is a polysaccharide of α-1,4-linked galacturonic acid (GalA) residues and is the predominant form of pectin in *A. thaliana* [3, 4]. It is synthesized at the Golgi apparatus and secreted from cells in a highly methyl-esterified form. These methyl ester groups can subsequently be removed by enzymatic activity in the cell wall, allowing for a range of methyl-esterification states. A broad range of HGA methyl-esterification patterns are recognized by the JIM7 antibody [5], and Figure 1A shows uniform JIM7 binding within the walls of guard cells, epidermal pavement, and mesophyll cells, indicating a wide distribution of HGA. HGA can form pectate calcium cross-links when continuous stretches of GalA residues are blockwise de-esterified, and this can be detected using the 2F4 antibody [6]. De-esterified, calcium cross-linked HGA was not detected in the guard cell walls or in neighboring cell walls but, instead, was limited to the junction regions between cells of the epidermis (Figure 1B). In contrast, relatively unesterified HGA (detected by antibody LM19) showed strong labeling in guard cell and epidermal cell walls (Figure 1C). Highly methyl-esterified HGA, as indicated by antibody LM20, was excluded from guard cells but was abundant in the junctions between guard cells and neighboring epidermal cells (Figure 1D). The LM20 signal was observed in the cell walls of epidermal pavement and mesophyll cells, as well as the cuticular ledges of guard cells. This specific pattern of pectins contrasted with the more uniform signal observed following immunolocalization with antibodies against other cell wall components, such as xyloglucan (LM25) (Figure 1E), while signal was absent when the primary antibody was not included (Figure 1F). Out of 36 cell wall antibodies tested, LM20 (against high methyl-esterified HGA) revealed a clear guard-cell-specific pattern (Figure S1; Table S1) being absent from guard cell walls but detected strongly in neighboring cell walls.

This is an open access article under the CC BY license (http://creativecommons.org/licenses/by/4.0/).

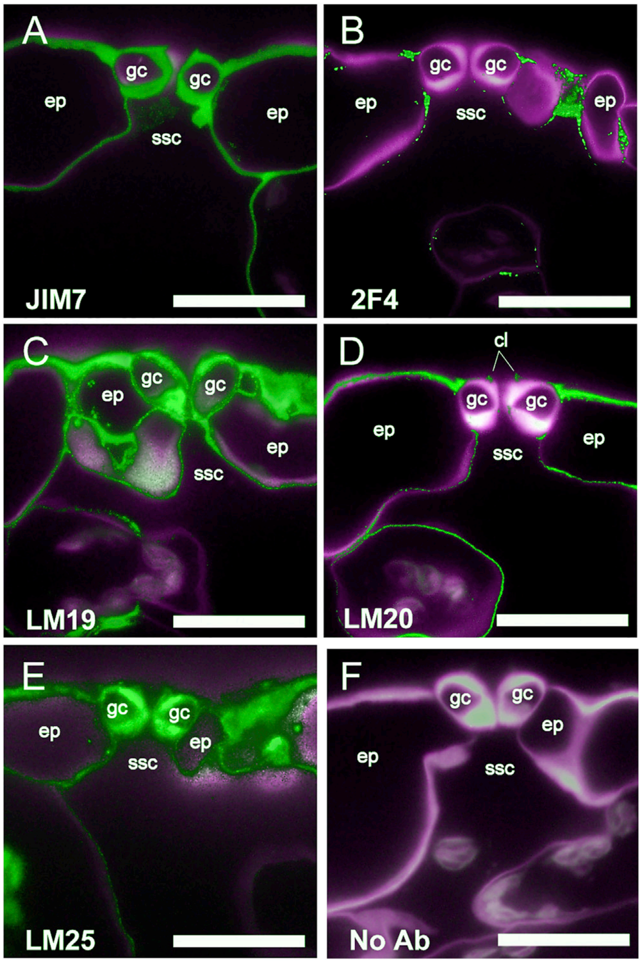

Figure 1. Guard Cells Show Specific Patterns of Wall Epitopes

(A) Ubiquitous presence of pectin in cell walls. The JIM7 antibody binds to HGA with a broad range of methyl-esterification and shows labeling in all cell walls in a cross-section through the epidermis (ep) encompassing guard cells (gc) above a sub-stomatal cavity (ssc).

(B) Calcium cross-linked HGA is restricted to cell interstices. The 2F4 antibody indicates cell walls containing calcium cross-linked HGA characterized by stretches of unesterified HGA residues.

(C) Unesterified HGA is present in GC walls. Binding of the LM19 antibody indicates that HGA with no or little esterification is prevalent in all cell walls of the epidermis.

(D) Highly methyl-esterified pectin is absent from the guard cell wall, as indicated by the lack of binding of the LM20 antibody.

(E) Binding of the LM25 antibody indicates that xyloglucan is present in all cell walls of the epidermis.

(F) A control with no primary antibody (Ab) showing low levels of autofluorescence against the Calcofluor White staining of the cell wall.

In all panels, the green signal shows binding of the specific primary antibody indicated, and the magenta signal (false color) indicates Calcofluor White fluorescence of cell walls. Scale bars, 20 μm. See also Figure S1 and Table S1.

These results can be compared with previous investigations of guard cell wall composition [7, 8]. For example, analysis of *Commelina communis* has shown that guard cells are rich in pectin but did not report on the differential methyl-esterification patterns described here. Our data show that both highly methyl-esterified and calcium cross-linked blockwise de-esterified HGA are excluded from the guard cells and that un-esterified HGA is the predominant form of pectin in the guard cell wall. Pectic arabinans have previously been implicated as being important for guard cell movements via ectopic application of carbohydrate-modifying enzymes [8]. Thus, our data support the idea that the structural properties of the pectin network are important for guard cell function. We took a molecular genetic approach to test this hypothesis.

Identification of a Mutant, *pme6-1*, with Altered Guard Cell Wall Pectin Distribution

The enzymes that modify plant cell wall pectins are typically encoded by large gene families. For example 66, 35, and 89 pectin methylesterase genes have been annotated in *A. thaliana*, *Oryza sativa*, and *Populus trichocarpa,* respectively [9]. The encoded proteins contain pectin methylesterase (PME), or both PME and pectin methylesterase inhibitor domains (proPME proteins). The PME and proPME enzymes control the methyl-esterification status of HGA by removing methyl ester groups from HGA [9]. This large number of genes has made it difficult to attribute specific pectin modifications of cell walls to particular physiological properties. As our experiments identified guard cells as having a distinct pectin methyl-esterification status, we sought to identify genes encoding pectin-modifying enzymes with a guard-cell-specific expression pattern. We focused on a proPME gene, *PME6* (TAIR: *AT1G23200*), which is expressed at >36-fold higher levels in guard cell protoplasts relative to mesophyll cell protoplasts [10] and is also expressed during seed coat development [11]. *PME6* expression has previously been shown to be repressed in *scap1*, a mutant with altered expression of cell wall modification genes and a resultant change in methyl-esterification state [12]. Negi et al. [12] proposed that *PME6* might act downstream of *SCAP1* to elicit at least part of the stomatal phenotype observed.

PME6 encodes a single PMEI domain and a PME domain that contains the two conserved active-site aspartic acid residues necessary for PME activity [9], as well as an N-terminal signal peptide, suggesting that it is a secreted protein. A *PME6* promoter β-glucuronidase (GUS) fusion construct containing approximately 1,400 bp upstream of the start codon (*proPME6::GUS*) was stably introduced into *A. thaliana*, and GUS histochemical localization indicated that this DNA region directs expression predominantly in mature guard cells (Figure S2A). Analysis of transcriptome data indicated that *PME6* mRNA accumulates to a high level in the *scrm-D* mutant, which has an excess of mature guard cells [13] and to a lower level in mutants in which epidermal cell differentiation is blocked at the pavement cell stage (*spch*) [14] or at the stage of meristemoid formation (*scrm-D mute*) [13, 15] (Figure S1B). These data suggest that *PME6* is expressed in guard cells at a relatively late stage of differentiation. Transcriptome data indicated that *PME6* is also expressed in siliques [16], and analysis of the *proPME6::GUS* lines confirmed this. Analysis of a *pme6* mutant (described later) did not reveal any change in seed germination or seed weight, so our further investigation focused on stomatal function. To investigate the function of *PME6*, we obtained an *A. thaliana* line with a Ds transposon inserted within the *PME6* gene (hereinafter referred to as *pme6-1*) from the Nottingham Arabidopsis Stock Centre. PCR and RT-PCR analyses showed that *pme6-1*

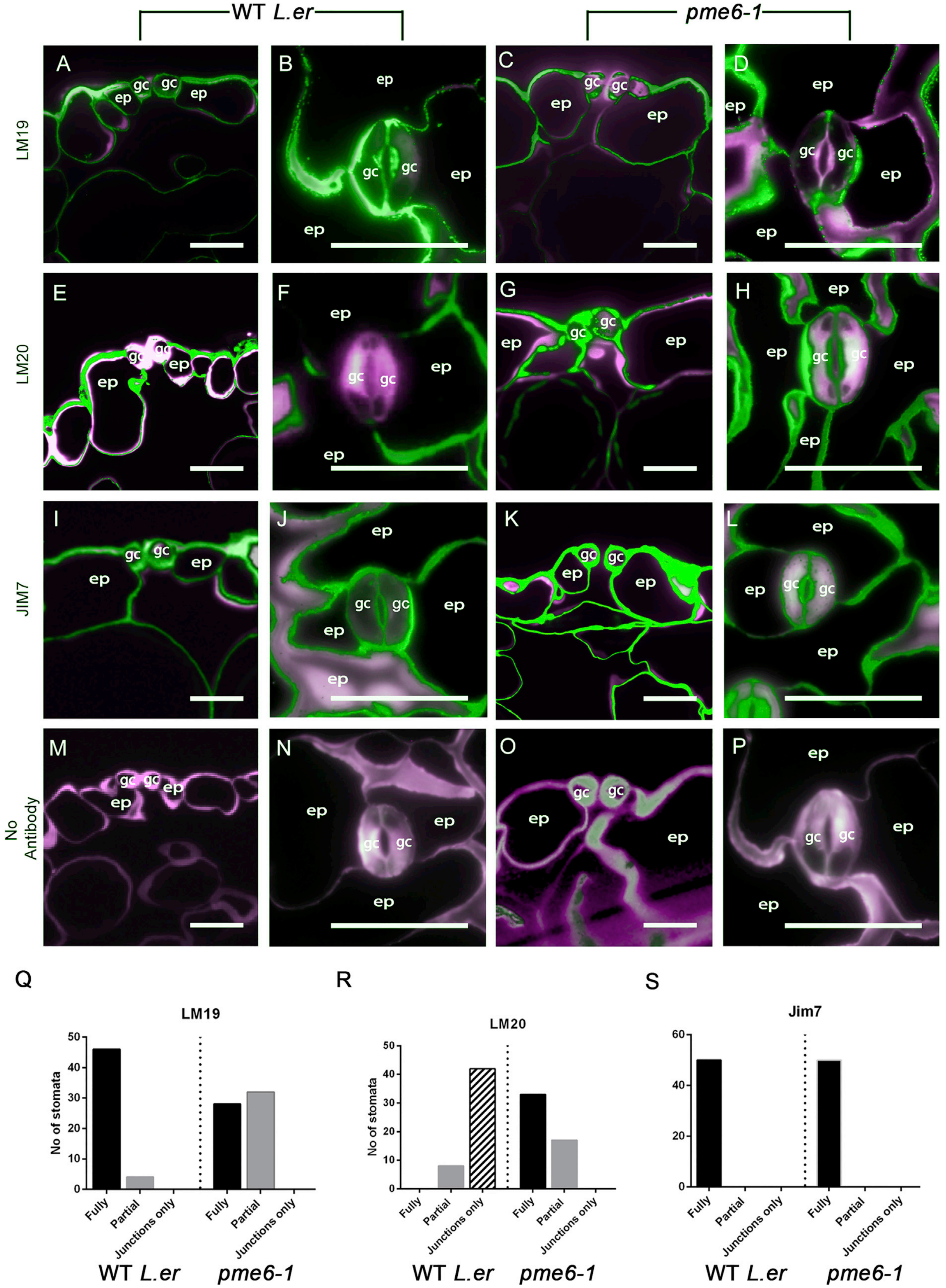

(legend on next page)

homozygous plants harbor an insertion in the single intron of the *PME6* gene (Figure S2C) and have no detectable expression of the *PME6* mRNA transcript (Figure S2D). Complementation of the *pme6-1* line by introducing a native *PME6* gene construct under the control of the *proPME6* promoter restored the levels of *PME6* mRNA in two independent lines (*proPME6::pme6*) (Figure S2D).

Immunolocalization analyses of *pme6-1* revealed a major change in the methyl-esterification status of guard cell wall pectins. We first confirmed that the guard cells of *L. erecta* wild-type (WT) plants (the *pme6-1* background) showed strong binding of LM19 (Figures 2A and 2B), indicating an abundance of relatively unesterified pectin. There was an absence of LM20 binding (Figures 2E and 2F), indicating that highly methyl-esterified pectin is absent from the guard cells. In contrast, *pme6-1* plants had a reduction in the levels of de-esterified pectin in the guard cell, as indicated by the weaker binding of LM19 (Figures 2C and 2D) and abundant highly esterified pectin, as indicated by LM20 binding (Figures 2G and 2H). These data indicate that the structure of the HGA component of the pectin network has been altered in the *pme6-1* knockout line and, in particular, that the pectin of guard cells is more highly methyl-esterified in plants lacking *PME6*. JIM7-binding patterns remained consistent between the WT and *pme6-1* (Figures 2I–2L), indicating that the differences observed in Figures 2A–2H were due to an alteration in the methyl-esterification status of the guard cells rather than a change in the overall distribution of HGA in the cell wall. The pattern of methyl-esterification in guard cells was highly reproducible, as shown in Figures 2Q–2S. *pme6-1* lines complemented with a *proPME6::PME6* construct showed a restoration of the WT methyl-esterification pattern (Figure S3). Analysis of controls (Figures 2M–2P) indicated that the patterns observed in Figures 2A–2L did not simply reflect patterns of cell wall thickness or overall distribution of cellulose. These data indicate that *PME6* is crucial for the de-methyl-esterification of guard cell wall HGA.

A Mutant with Altered Guard Cell Wall Pectin Methylation Has Impaired Stomatal Function

To study the functional significance of the changing pectin methyl-esterification status of the guard cells, we investigated the stomatal opening and closure responses of *pme6-1*. Figure 3A shows the stomatal aperture response in isolated epidermal strips exposed to buffers supplied with elevated (1,000 ppm) or decreased (0 ppm) levels of CO_2. Exposure to elevated CO_2 caused WT stomatal apertures to decrease, and CO_2-free air caused WT apertures to increase, as previously reported [17]. In contrast, *pme6-1* stomatal apertures were relatively insensitive to CO_2, with the responses to both elevated and decreased CO_2 being lost. The stomatal aperture response to CO_2 was restored in the complemented lines. A restricted ability of *pme6-1* stomata to respond to abscisic acid, a classical regulator of stomatal function [17], was also observed (Figure S4E), suggesting that the altered pectin methyl-esterification status of the guard cells was affecting a fundamental property of the stomata.

Since stomata play a major role in controlling the water relations of the plant, thermal imaging was used to investigate the effects of the *pme6-1*-altered guard cell wall properties at the whole-plant level by gauging leaf temperature as a measure of evaporative cooling (which is tightly linked to stomatal function [18]). Under well-watered growth conditions, there were minimal differences in temperature between the *pme6-1*, the WT, and complemented mutant lines (Figure 3B). However, under drought conditions, *pme6-1* plants were significantly cooler than the WT and complemented lines (Figures 3B and 3C), probably due to a higher rate of transpiration through their more open stomata. These results are consistent with the data in Figure 3A, indicating that the *pme6-1* mutant stomata have a more restricted range of stomatal opening/closure as a result of altered guard cell wall properties and a more restricted response to ABA (Figure S4). To further investigate the physiological outcome of altered stomatal performance in the *pme6-1* mutant, we conducted infrared gas exchange analysis to assess stomatal conductance (g_s), in response to shifts in CO_2 conditions. Under ambient CO_2 conditions, the *pme6-1* leaves had a higher g_s compared to the WT (Figure 4A). When exposed to elevated CO_2, the *pme6-1* g_s value decreased slightly but remained higher than the WT value. However, when the CO_2 level was decreased to sub-ambient, both *pme6-1* and WT g_s increased but the maximal level achieved by the *pme6-1* leaves plateaued at a lower level than that of the WT; thus, the overall dynamic range of g_s shown by the *pme6-1* leaves was lower than for the WT. Unlike stomatal aperture measurements in epidermal peels, g_s is influenced not only by the guard cells but also by a variety of physiological processes in the whole leaf and plant, which may counteract the actions of individual stomata, leading to an amelioration of the response seen in isolated peels [19]. The data in Figure 4A indicate that *pme6-1* stomata have a more restricted dynamic range of opening/closure

Figure 2. Guard Cell Wall Pectin Composition Is Altered in *pme6-1* Plants

(A–D) The high level of unesterified HGA in WT guard cells indicated by LM19 antibody binding in both cross-sections (A) and paradermal sections (B) is greatly diminished in *pme6-1* (C and D). ep, epidermis; gc, guard cells.

(E–H) Highly methyl-esterified HGA is absent in WT guard cell walls (E and F) but accumulates in the guard cell walls of the *pme6-1* mutant, as revealed by binding of the LM20 antibody (G and H).

(I–L) The general distribution of HGA (indicated by the JIM7 antibody) is similar in the WT (I and J) and the *pme6-1* mutant (K and L).

(M–P) Control sections not hybridized with primary antibody but stained with Calcofluor White indicate the signal specificity of the immunolabeling experiments in (A)–(L) and the general distribution of the cell wall material. In all panels, the green signal shows binding of the specific primary antibody indicated, and the magenta signal (false color) indicates Calcofluor White fluorescence of cell walls.

(Q–S) Counting of stomata showing the patterns of labeling with each antibody indicate the switch in LM20/LM19 labeling pattern between WT and the *pme6-1* mutant guard cells. Localization of fluorescence in transverse sections after antibody binding was scored as fully covering guard cells (as in I), partially covering guard cells (as in C), or limited to guard cell-epidermal cell junctions (as in E). Data are shown for LM19 (Q), LM20 (R), and Jim7 (S) immunolabeling. Quantification was based on scoring patterns from 50 stomata, with five stomata scored from each of ten plants.

Scale bars, 20 μm. See also Figures S1–S3.

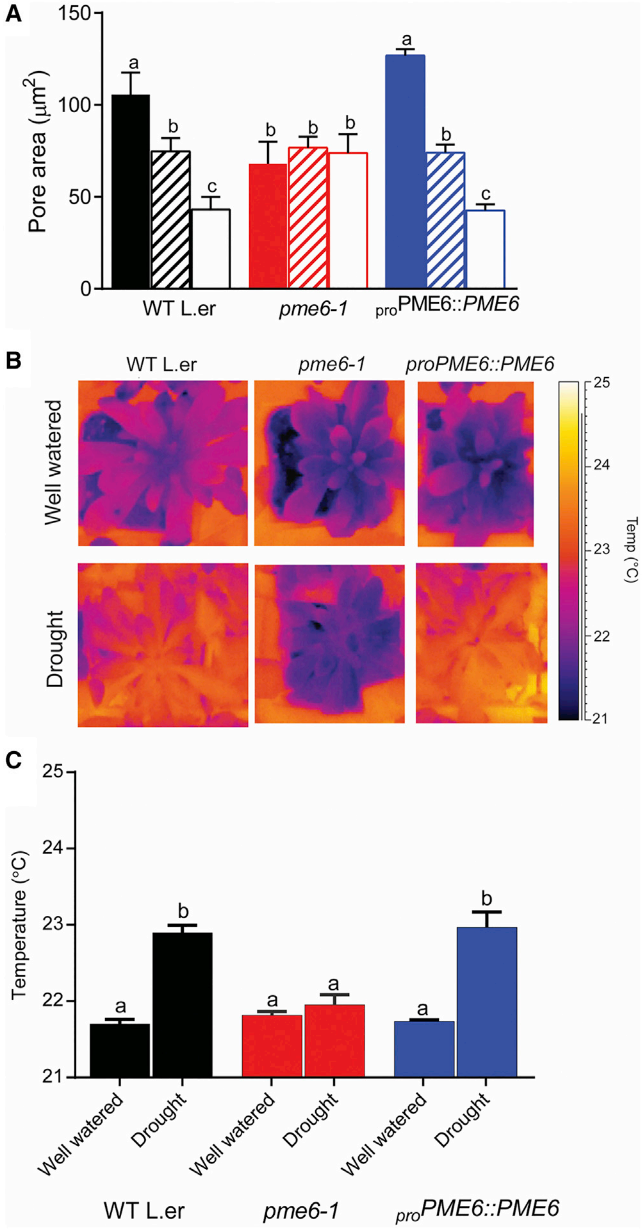

Figure 3. *pme6-1* Plants Have Altered Guard Cell Physiology and Water Relationships

(A) Guard cell opening/closure response to changing CO_2 concentration is lost in the *pme6-1* mutant. Pore area was measured from stomata in epidermal peels taken from the genotypes indicated (WT, *pme6-1*, and *pme6-1* complemented with a $_{pro}$PME6::PME6 construct) after incubation of the peels with either CO_2-free air (0 ppm CO_2; solid bars), ambient CO_2 (hatched bars) or high CO_2 (1,000 ppm; open bars). Each column shows the mean and SEM. (n = 6), with statistical differences determined by ANOVA with a post hoc Tukey test. Columns indicated with identical letters cannot be distinguished from each other ($p < 0.01$).

(B) *pme6-1* plants are less able to adjust leaf temperature under drought conditions. Thermal images are shown of well-watered plants of the genotypes indicated (top images) taken at day 0 post-drought. Images of equivalent plants at day 5 post-drought (lower panel) show that the *pme6-1* plants have a lower leaf temperature than the WT or the complemented *pme6-1* mutant.

(C) Quantification of thermal image data shows that *pme6-1* leaf temperature does not change significantly under drought conditions, while the WT and the complemented mutant leaf temperature increases. Each bar represents the mean temperature for the rosette with error bars indicating SEM (n = 6). Statistical differences were determined by ANOVA with a post hoc Tukey test. Columns indicated with identical letters cannot be distinguished from each other ($p < 0.05$).

See also Figure S4.

that cannot be completely compensated for at the whole plant level, thus leading to altered water relationships.

An altered dynamic range of guard cell swelling and deflation was also indicated by direct observation of stomatal pore area after immersion in an osmoticum expected to decrease turgor pressure [8]. Despite being subjected to a similar decrease in osmotic potential (1.23 MPa), the *pme6-1* stomatal pores remained significantly larger than those of the WT (Figure 4B). Analysis of guard cells by electron microscopy did not reveal any overt difference in surface shape or size (Figures S2E and S2F), and overall cell ultrastructure appeared similar in the two genotypes (Figures S2G and S2H), suggesting that the different behavior of the stomata was not due to large-scale change in cell structure but rather to some alteration in the mechanical properties of the cell wall. The differential response to a similarly imposed biophysical challenge via mannitol treatment supports the idea that the pectin structure in the guard cells sets the mechanics of the cellular complex, thus limiting the range of size change possible. It has been postulated that the methyl-esterification status of pectin influences the ability of HGA domains to associate via Ca^{2+} cross-links and that the degree of Ca^{2+} cross-linking has a major effect on the mechanical stiffness of the cell wall matrix. Indeed, previous work on guard cell walls suggested that arabinan side chains of the rhamnogalacturonan-I pectic domain associated with HGA might play a role in physically separating HGA domains, thus influencing Ca^{2+} cross-linking [8]. Since the formation of pectic network structures will be strongly dependent on the methyl-esterification status of HGA, PMEs can be predicted to play a major role in determining the overall mechanical properties of the cell wall. However, although recent evidence strongly supports the role of pectin methyl-esterification status in cell wall mechanics [20–22], simple inference of mechanics based on pectin methyl-esterification has proven problematic, since it appears to be highly dependent on cellular context [23, 24]. Our data suggest a situation in which a reduction in pectin de-esterification leads to guard cells that are relatively stiffer, limiting potential changes in cell size.

The Influence of Altered Guard Cell Wall Structure on Plant Growth Is Environment Dependent

From our gas exchange analysis, we derived A/Ci (photosynthesis rate/internal CO_2 concentration) curves relating instantaneous carbon assimilation rate to CO_2 level (Figure 4D). At ambient CO_2, both *pme6-1* and WT leaves showed similar assimilation rates; however, at elevated CO_2, a much higher rate was measured in *pme6-1*. As CO_2 level rises relative to O_2, it is expected that photorespiration in C3 plants (such as *Arabidopsis*) will decrease [25], thus leading to a higher net assimilation rate. Leaves with stomata that show a decreased closure response to CO_2, as observed here for *pme6-1*, might be expected to have higher internal CO_2 levels and, thus, a greater increase in assimilation rate than the WT, as indicated

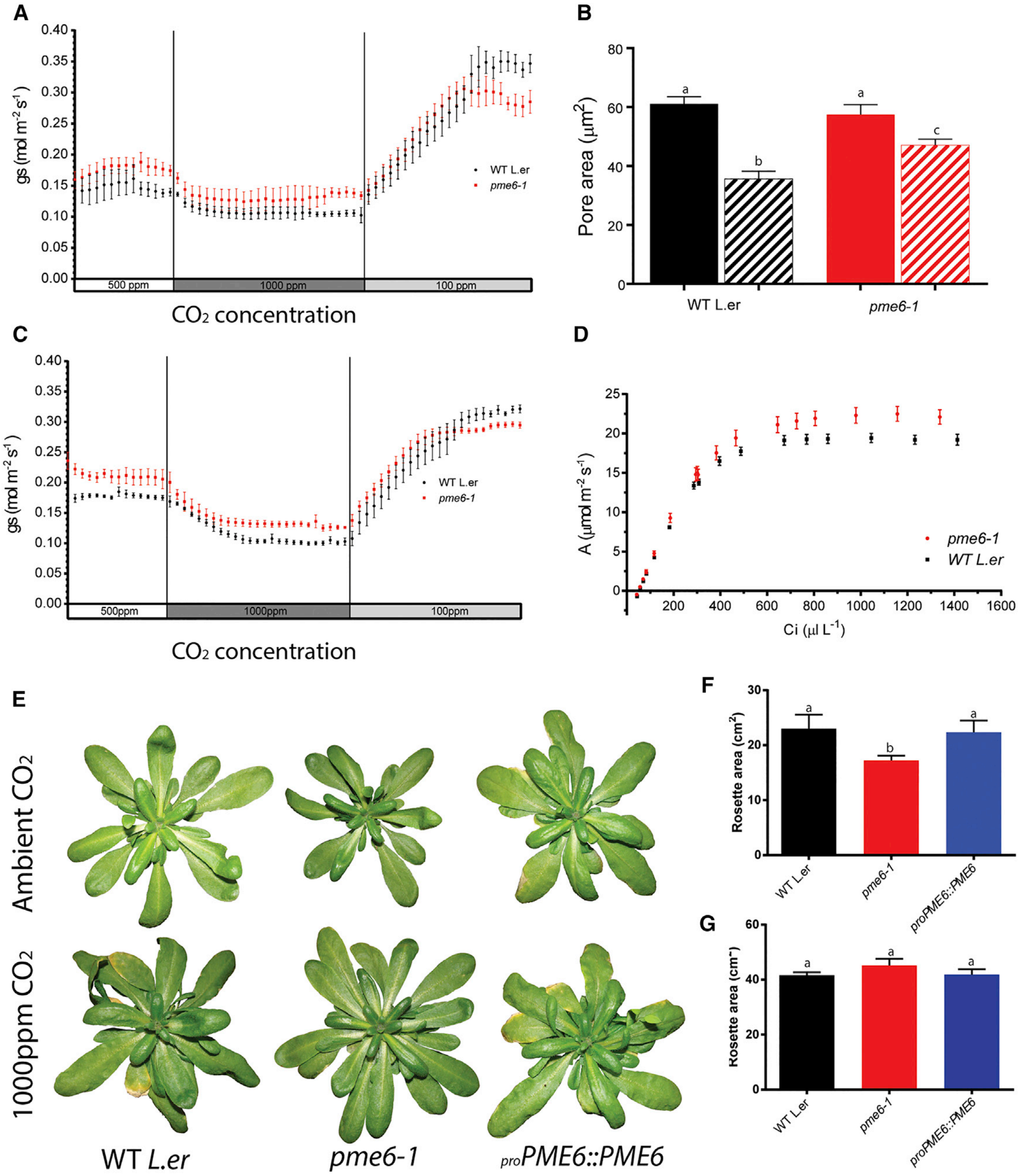

Figure 4. *pme6-1* Plants Show a Limited Dynamic Range of Stomatal Movement and Decreased Growth under Ambient CO_2, which Is Rescued by Elevated CO_2

(A) *pme6-1* leaves show a limited dynamic range in stomatal conductance (g_s) in response to changing CO_2 level. Gas exchange data for WT and *pme6-1* leaves show that, under ambient CO_2 conditions, the *pme6-1* leaves have higher g_s than the WT. Following exposure to elevated (1,000 ppm) CO_2, g_s in both mutants and WTs fall. Exposure to a low (100-ppm) CO_2 regime induces increased g_s, but the *pme6-1* g_s trace plateaus to a lower value than for WT leaves. Error bars indicate the SEM (n = 8).

(B) *pme6-1* stomata show a differential pore size response after incubation in high osmoticum. Stomatal pore areas were measured in epidermal peels from either WT or *pme6-1* leaves incubated either in resting buffer (solid bars) or resting buffer with addition of mannitol to 0.5 M (hatched bars). Statistical differences were determined by ANOVA and a post hoc Tukey test. Columns indicated with identical letters cannot be distinguished from each other ($p < 0.01$, n = 3, with 40 stomata counted from a total of four plants, repeated on 3 consecutive days). Error bars indicate the SEM.

(C) The more limited dynamic range in g_s exhibited by *pme6-1* leaves is maintained after the growth of plants at elevated CO_2. Gas exchange data for WT and *pme6-1* leaves taken from plants grown continually under elevated CO_2. The traces for the WT and *pme6-1* as the CO_2 level is altered during gas exchange analysis are comparable to those shown in (A), with the *pme6-1* trace again reaching a lower plateau after exposure to sub-ambient CO_2 level (n = 8).

(D) At elevated CO_2 levels, the *pme6-1* leaves have a greater potential to assimilate CO_2 than WT leaves. A/Ci curve analysis of WT and *pme6-1* leaves indicates that the instantaneous C assimilation rate at ambient CO_2 levels is comparable but that as C_i increases, the *pme6-1* leaves show a greater maximum potential assimilation rate (n = 5 for WT; n = 6 for *pme6-1*; error bars indicate the SEM).

(legend continued on next page)

in Figure 4D. To investigate the outcome of this at a whole-plant level, we compared the growth of WT and *pme6-1* mutant plants under ambient and elevated CO_2. *pme6-1* plants were smaller than WTs when grown under ambient CO_2 (Figures 4E and 4F). When grown under elevated CO_2, the *pme6-1* and WT plants were larger than the equivalent plants grown under ambient CO_2 (as expected) [26], but there was no difference in size between mutants and WTs (Figures 4E and 4G). Gas exchange analysis confirmed that the more limited dynamic range in g_s observed in the *pme6-1* leaves under ambient CO_2 conditions was maintained when the plants were grown under elevated CO_2 (Figure 4C), indicating that the underlying, more limited, dynamic range of stomatal function was also present in the mutant under these growth conditions, as expected for a genetically determined change in cell wall structure.

Stomatal size and density are well-characterized parameters known to influence leaf performance under various environments [26]. In the wake of rising atmospheric CO_2 levels, there has been much interest in understanding how stomatal parameters provide an insight into past evolutionary events linked to earlier environments [27] and into the potential modification of stomata to create crop plants better attuned to present and predicted climates [28, 29]. The potential role of the guard cell wall in setting and modulating the response dynamics of stomata has been underexplored. Since our analysis did not indicate any differences in stomatal density or stomatal index between *pme6-1* and WT leaves (Figures S4A–S4D), the most plausible interpretation of our data is that that modulation of the pectin matrix of guard cells leads to altered wall properties so that the stomata are mechanically limited in their responses to exogenous cues, thus altering plant-water relations.

Despite a firm theoretical basis for the importance of differential guard cell wall stiffness in the mechanism of stomata opening and closure in response to altered turgor pressure [1, 30], experimental evidence has often been correlative, e.g., measurements of cell wall thickening, observations of cellulose microfibril orientation [2]. Indeed, despite a wealth of physiological data on turgor pressure and ion fluxes [31], and intricate details of molecular signaling in guard cells [17], the causal relationship between guard cell wall structure/composition and stomatal function has been surprisingly underexplored. The most insightful data have come from experiments in which the exogenous supply of cell-wall-modifying enzymes suggested an important role for pectic arabinans in stomatal function [8]. However, the nature of the genes involved and, indeed, formal genetic evidence to support this hypothesis, are lacking. In this paper, we provide molecular data to show that not only do *Arabidopsis* guard cells have a specific HGA methyl-esterification status (unesterified), but also that this status is required for normal stomatal function. The simplest interpretation of our data is that abnormal pectin methyl-esterification alters mechanical properties of the guard cells, leading to an inability to show appropriate opening and closure in response to environmental signals. Due to the importance of the mechanical properties of guard cells in setting the dynamics of stomatal opening and, thus, whole-plant/water relations, this alteration in guard-cell-specific cell wall gene expression leads to a poorer ability of the leaf to control water loss under drought conditions and poorer growth of the plants under ambient CO_2 levels. Interestingly, this growth defect was overcome when the mutant plants were grown at elevated CO_2, indicating that, under certain conditions, altered guard cell wall mechanics are not detrimental. In summary, our data indicate that, in addition to the well-explored regulation of stomatal dynamics via signal transduction signals acting on ion transport to vary turgor pressure, cell wall modification plays an important role in setting the overall limits of the system. Although this study focused on the role of pectins, it is clear that cell wall matrix components function together to influence mechanical properties [32], and exploring the roles of both structural carbohydrates, such as xyloglucans and cellulose [33], and modulating protein factors, such as expansins [34], will allow a deeper understanding of the system. Allied to this, targeted modulation of guard cell wall structure provides a novel avenue for the future manipulation of stomatal function.

EXPERIMENTAL PROCEDURES

Details of the experimental procedures are available in the Supplemental Information.

SUPPLEMENTAL INFORMATION

Supplemental Information includes Supplemental Experimental Procedures, four figures, and one table and can be found with this article online at http://dx.doi.org/10.1016/j.cub.2016.08.021.

AUTHOR CONTRIBUTIONS

Conceptualization, A.J.F. and J.E.G.; Investigation, S.A., L.H., N.E., A.B., M.L., Y.V, and A.J.F.; Writing – Original Draft, A.J.F., J.E.G., and S.A.; Writing – Review & Editing, A.J.F., J.E.G., S.A., L.H., J.P.K., Y.V., and H.V.S.; Resources, J.P.K.; Supervision, A.J.F. and J.E.G.; Funding Acquisition, A.J.F., J.E.G., and H.V.S.

ACKNOWLEDGMENTS

The work reported here was funded by a White Rose BBSRC-DTP award (to S.A. and A.J.F.); BBSRC grant BB/I002154/1 (to L.H. and J.E.G.); the Gatsby Foundation (to A.B.), the U.S. Department of Energy, Office of Science, Office of Biological and Environmental Research, through Contract DE-AC02-05CH11231 between the Lawrence Berkeley National Lab and the U.S. Department of Energy (to Y.V. and H.V.S.); and a Leverhulme Research Fellowship (to A.J.F.). Ray Wightman (SLCU, Cambridge) assisted with SEM. The Microscopy Facility at the Sainsbury Laboratory is supported by the Gatsby Charitable Foundation.

Received: March 15, 2016
Revised: July 6, 2016
Accepted: August 5, 2016
Published: October 6, 2016

(E–G) *pme6-1* plants are smaller than WTs under ambient CO_2, but growth at elevated CO_2 leads to plants attaining a similar size. Images of plants (genotypes as indicated) under ambient CO_2 are shown in (E, top row) and under elevated (1,000 ppm) CO_2 in (E, bottom row). Quantitation of total rosette area of plants grown under ambient CO_2 (F) shows that *pme6-1* plants achieve a smaller final size, whereas growth in elevated CO_2 (G) leads to all plants reaching a similar mean size. In (F and G), error bars indicate the SEM, n = 8.
See also Figure S4.

CellPress

REFERENCES

1. Franks, P.J., Cowan, I.R., and Farquhar, G.D. (1998). A study of stomatal mechanics using the cell pressure probe. Plant Cell Environ. *21*, 94–100.
2. Palevitz, B.A., and Hepler, P.K. (1976). Cellulose microfibril orientation and cell shaping in developing guard cells of Allium: The role of microtubules and ion accumulation. Planta *132*, 71–93.
3. Zablackis, E., Huang, J., Müller, B., Darvill, A.G., and Albersheim, P. (1995). Characterization of the cell-wall polysaccharides of Arabidopsis thaliana leaves. Plant Physiol. *107*, 1129–1138.
4. Caffall, K.H., and Mohnen, D. (2009). The structure, function, and biosynthesis of plant cell wall pectic polysaccharides. Carbohydr. Res. *344*, 1879–1900.
5. Verhertbruggen, Y., Marcus, S.E., Haeger, A., Ordaz-Ortiz, J.J., and Knox, J.P. (2009). An extended set of monoclonal antibodies to pectic homogalacturonan. Carbohydr. Res. *344*, 1858–1862.
6. Liners, F., Letesson, J.J., Didembourg, C., and Van Cutsem, P. (1989). Monoclonal antibodies against pectin: recognition of a conformation induced by calcium. Plant Physiol. *91*, 1419–1424.
7. Majewska-Sawka, A., Münster, A., and Rodríguez-García, M.I. (2002). Guard cell wall: immunocytochemical detection of polysaccharide components. J. Exp. Bot. *53*, 1067–1079.
8. Jones, L., Milne, J.L., Ashford, D., and McQueen-Mason, S.J. (2003). Cell wall arabinan is essential for guard cell function. Proc. Natl. Acad. Sci. USA *100*, 11783–11788.
9. Pelloux, J., Rustérucci, C., and Mellerowicz, E.J. (2007). New insights into pectin methylesterase structure and function. Trends Plant Sci. *12*, 267–277.
10. Yang, Y., Costa, A., Leonhardt, N., Siegel, R.S., and Schroeder, J.I. (2008). Isolation of a strong Arabidopsis guard cell promoter and its potential as a research tool. Plant Methods *4*, 6.
11. Levesque-Tremblay, G., Müller, K., Mansfield, S.D., and Haughn, G.W. (2015). HIGHLY METHYL ESTERIFIED SEEDS is a pectin methyl esterase involved in embryo development. Plant Physiol. *167*, 725–737.
12. Negi, J., Moriwaki, K., Konishi, M., Yokoyama, R., Nakano, T., Kusumi, K., Hashimoto-Sugimoto, M., Schroeder, J.I., Nishitani, K., Yanagisawa, S., and Iba, K. (2013). A Dof transcription factor, SCAP1, is essential for the development of functional stomata in Arabidopsis. Curr. Biol. *23*, 479–484.
13. Kanaoka, M.M., Pillitteri, L.J., Fujii, H., Yoshida, Y., Bogenschutz, N.L., Takabayashi, J., Zhu, J.-K., and Torii, K.U. (2008). SCREAM/ICE1 and SCREAM2 specify three cell-state transitional steps leading to arabidopsis stomatal differentiation. Plant Cell *20*, 1775–1785.
14. MacAlister, C.A., Ohashi-Ito, K., and Bergmann, D.C. (2007). Transcription factor control of asymmetric cell divisions that establish the stomatal lineage. Nature *445*, 537–540.
15. Pillitteri, L.J., Sloan, D.B., Bogenschutz, N.L., and Torii, K.U. (2007). Termination of asymmetric cell division and differentiation of stomata. Nature *445*, 501–505.
16. Schmid, M., Davison, T.S., Henz, S.R., Pape, U.J., Demar, M., Vingron, M., Schölkopf, B., Weigel, D., and Lohmann, J.U. (2005). A gene expression map of Arabidopsis thaliana development. Nat. Genet. *37*, 501–506.
17. Kim, T.-H., Böhmer, M., Hu, H., Nishimura, N., and Schroeder, J.I. (2010). Guard cell signal transduction network: advances in understanding abscisic acid, CO2, and Ca2+ signaling. Annu. Rev. Plant Biol. *61*, 561–591.
18. Wang, Y., Holroyd, G., Hetherington, A.M., and Ng, C.K.Y. (2004). Seeing 'cool' and 'hot'–infrared thermography as a tool for non-invasive, high-throughput screening of Arabidopsis guard cell signalling mutants. J. Exp. Bot. *55*, 1187–1193.
19. Pantin, F., Monnet, F., Jannaud, D., Costa, J.M., Renaud, J., Muller, B., Simonneau, T., and Genty, B. (2013). The dual effect of abscisic acid on stomata. New Phytol. *197*, 65–72.
20. Peaucelle, A., Braybrook, S.A., Le Guillou, L., Bron, E., Kuhlemeier, C., and Höfte, H. (2011). Pectin-induced changes in cell wall mechanics underlie organ initiation in Arabidopsis. Curr. Biol. *21*, 1720–1726.
21. Peaucelle, A., Louvet, R., Johansen, J.N., Höfte, H., Laufs, P., Pelloux, J., and Mouille, G. (2008). Arabidopsis phyllotaxis is controlled by the methyl-esterification status of cell-wall pectins. Curr. Biol. *18*, 1943–1948.
22. Peaucelle, A., Braybrook, S., and Höfte, H. (2012). Cell wall mechanics and growth control in plants: the role of pectins revisited. Front. Plant Sci. *3*, 121.
23. Hongo, S., Sato, K., Yokoyama, R., and Nishitani, K. (2012). Demethylesterification of the primary wall by PECTIN METHYLESTERASE35 provides mechanical support to the Arabidopsis stem. Plant Cell *24*, 2624–2634.
24. Liu, Q., Talbot, M., and Llewellyn, D.J. (2013). Pectin methylesterase and pectin remodelling differ in the fibre walls of two gossypium species with very different fibre properties. PLoS ONE *8*, e65131.
25. Long, S.P., Ainsworth, E.A., Rogers, A., and Ort, D.R. (2004). Rising atmospheric carbon dioxide: plants FACE the future. Annu. Rev. Plant Biol. *55*, 591–628.
26. Doheny-Adams, T., Hunt, L., Franks, P.J., Beerling, D.J., and Gray, J.E. (2012). Genetic manipulation of stomatal density influences stomatal size, plant growth and tolerance to restricted water supply across a growth carbon dioxide gradient. Philos. Trans. R. Soc. Lond. B Biol. Sci. *367*, 547–555.
27. Chater, C., Gray, J.E., and Beerling, D.J. (2013). Early evolutionary acquisition of stomatal control and development gene signalling networks. Curr. Opin. Plant Biol. *16*, 638–646.
28. Hepworth, C., Doheny-Adams, T., Hunt, L., Cameron, D.D., and Gray, J.E. (2015). Manipulating stomatal density enhances drought tolerance without deleterious effect on nutrient uptake. New Phytol. *208*, 336–341.
29. Franks, P.J.W., Doheny-Adams, T.W., Britton-Harper, Z.J., and Gray, J.E. (2015). Increasing water-use efficiency directly through genetic manipulation of stomatal density. New Phytol. *207*, 188–195.
30. Mott, K.A., and Franks, P.J. (2001). The role of epidermal turgor in stomatal interactions following a local perturbation in humidity. Plant Cell Environ. *24*, 657–662.
31. Pandey, S., Zhang, W., and Assmann, S.M. (2007). Roles of ion channels and transporters in guard cell signal transduction. FEBS Lett. *581*, 2325–2336.
32. Braybrook, S.A., and Jönsson, H. (2016). Shifting foundations: the mechanical cell wall and development. Curr. Opin. Plant Biol. *29*, 115–120.
33. Rui, Y., and Anderson, C.T. (2016). Functional analysis of cellulose and xyloglucan in the walls of stomatal guard cells of Arabidopsis thaliana. Plant Physiol. *170*, 1398–1419.
34. Goh, H.-H., Sloan, J., Dorca-Fornell, C., and Fleming, A. (2012). Inducible repression of multiple expansin genes leads to growth suppression during leaf development. Plant Physiol. *159*, 1759–1770.

Current Biology, Volume *26*

Supplemental Information

Stomatal Function Requires Pectin

De-methyl-esterification of the Guard Cell Wall

Sam Amsbury, Lee Hunt, Nagat Elhaddad, Alice Baillie, Marjorie Lundgren, Yves Verhertbruggen, Henrik V. Scheller, J. Paul Knox, Andrew J. Fleming, and Julie E. Gray

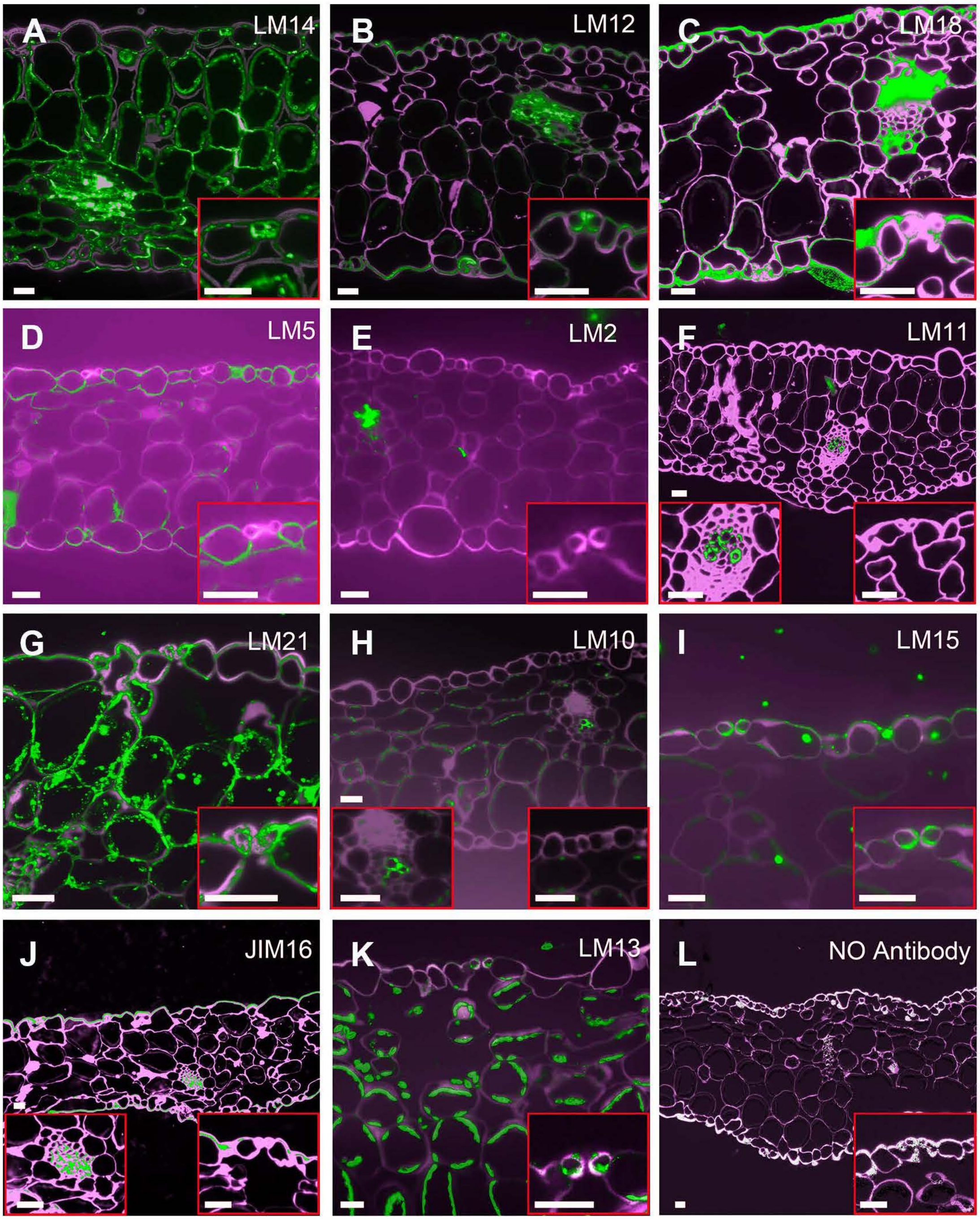

Figure S1. Related to Figure 1. A range of cell wall epitopes patterns are detected in the leaf by immunolabelling. (A) LM14 signal (green) indicates broad distribution of arabinogalactan protein in the mesophyll, epidermis and guard cells. (B) LM12 indicates feruloylated polymers are present in guard cells and vascular cells, with low levels of epidermal cell binding also present. (C) LM18 binding, which detects homogalacturonan which is partially methylesterified, was observed in the vasculature and the epidermis but not the guard cells. (D) LM5 binds (1-4)-β-D-Galactan and signal was detected in epidermal cells and sporadically in the mesophyll, with no guard cell binding observed. (E) LM2 binds an epitope of arabinogalactan proteins and was only detected in the vasculature. (F) LM11 binds xylan and arabinoxylan and was only detected in the vasculature. (G) LM21 recognises heteromannan. Strong signal was observed in the mesophyll with weaker but consistent binding in the epidermis and guard cells. (H) LM10 binds heteroxylan and was only detected in the vasculature. Signal in chloroplasts reflects autofluorescence (I) LM15 binds the XXXG motif of xyloglucan and binding was observed weakly in the mesophyll and epidermal cells and strongly in the guard cells. (J) JIM16 binds AGPs and was observed in the mesophyll and weakly in the epidermis but not in the guard cells. (K) LM13 binds arabinan and signal was observed in the guard cells. Signal in the chloroplasts reflects autofluorescence (L) Samples with no primary antibody show a low level of fluorescence in the green channel. Insets in A-L show detail either of guard cells (lower right in respective panel) or vasculature (lower left in respective panel). In all panels the green signal shows antibody signal following binding of the specific primary antibody indicated and the magenta signal (false-colour) indicates Calcofluor White fluorescence of cell walls.Scale bars = 20 µm.

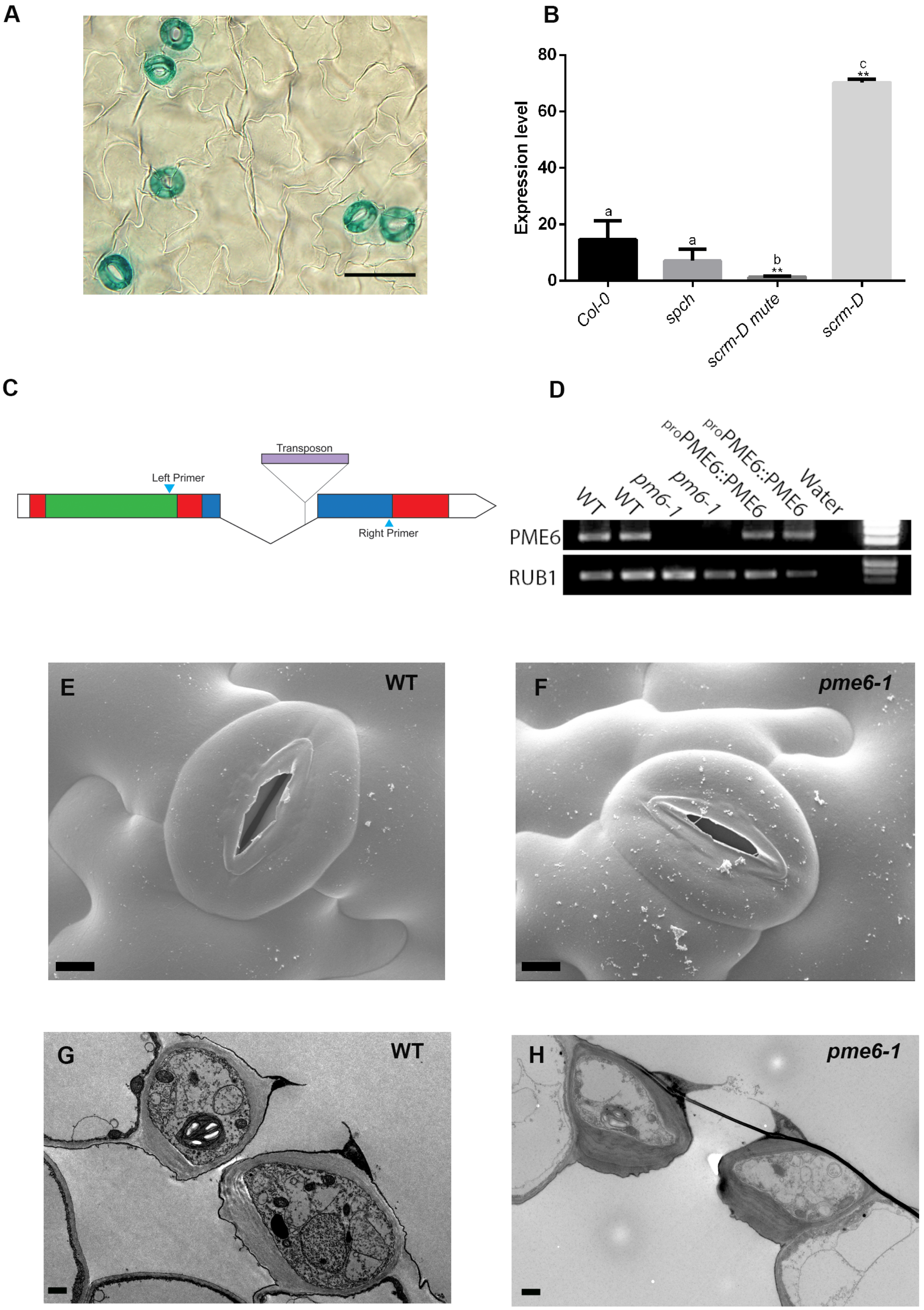
A
B
Expression level
80
60
40
20
0
a
a
b
**
c
**
Col-0
spch
scrm-D mute
scrm-D
C
Transposon
Left Primer
Right Primer
D
WT
WT
pm6-1
pm6-1
proPME6::PME6
proPME6::PME6
Water
PME6
RUB1
E
WT
F
pme6-1
G
WT
H
pme6-1

Figure S2

Figure S2. Related to Figure 2. *PME6* expression is up-regulated in guard cells but loss of *PME6* does not lead to a major change in guard cell shape and structure. (A) Histochemical staining of Arabidopsis leaves containing a $_{pro}$PME::GUS construct. Signal (blue) is observed only in mature guard cells. (B) *PME6* transcript levels are relatively high in the *scrm-D* mutant which has an increased number of mature stomata whereas *PME6* transcript levels are relatively low in mutants with an increased number of pavement cells (*spch*) or meristemoids (*scrm-D mute*). Columns indicate mean values, error bars = s.e.m. (n=5). Expression levels indicated with the same letter cannot be distinguished from each other at the 0.05 confidence limit (ANOVA and post-hoc Tukey, n= XX). (C) Schematic of the *pme6-1* locus containing a DS insertion mutant. The insertion is in an intron (blue) within the 3'UTR (red). Left and right primer sites used for PCR are indicated. (D) RT-PCR analysis of *PME6* transcript level in WT (*Landsberg erecta*), *pme6-1* mutant, and complemented *pme6-1* mutant lines. No transcript was detected in the *pme6-1* mutant line. Transcripts were detected in all samples using primers for the control gene *RUB1*. (E) cryoSEM image of a wild-type stomate and (F) a *pme6-1* stomate. (G) TEM of a wild-type stomata and (H) a *pme6-1* stomate. Scale bars = 25 µm in A; 4µm in E,F; 2 µm in G,H.

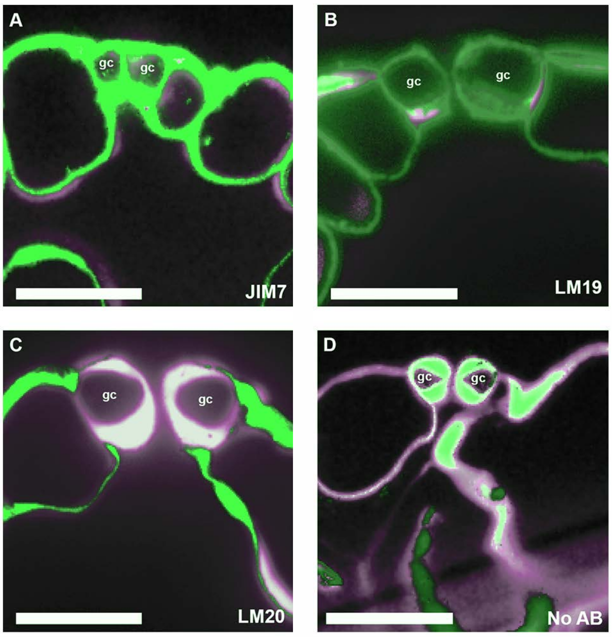

Figure S3. Related to Figure 2. Pectin methylation pattern in guard cells is restored to WT in complemented *pme6-1* plants. (A) JIM7 antibody labelling of the epidermis of the *pme6-1* mutant complemented with the $_{pro}$PME6::PME6 construct reveals that HGA is present in all cell walls. (B) Relatively un-esterified HGA, revealed using the LM19 antibody, is present in all cell walls of the complemented mutant, including those of the guard cells (C) Methyl esterified HGA, revealed using the LM20 antibody, is excluded from the guard cell wall in a pattern similar to that observed in WT plants (compare Fig 2E). (D) Controls with no primary antibody reveal a low level of autofluorescence. Antibody binding is indicated by green signal whereas magenta signal indicates Calcofluor staining of the cell wall. Observed patterns were consistent when replicated (n=8). Guard cells indicated by gc. Scale bars represent 20 µm.

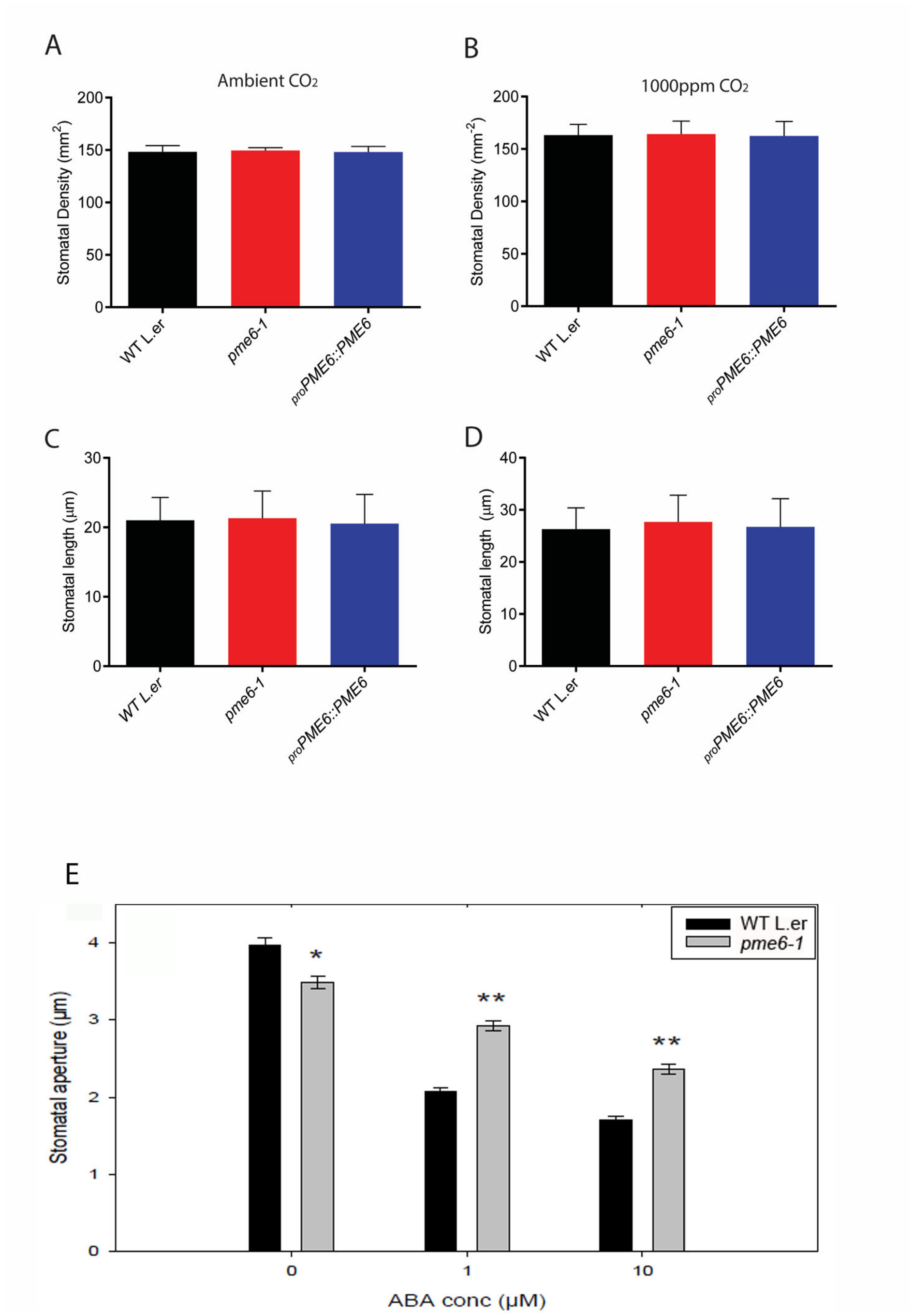

Figure S4. Related to Figures 3 and 4. *pme6-1* stomata are less responsive to ABA induced closure but stomatal size and density are unchanged (A) Stomatal density in leaves from WT, *pme6-1* and *pme6-1* complemented plants grown under ambient CO2 or (B) elevated (1000 ppm) CO2. (C) Stomatal length in leaves from WT, *pme6-1* and *pme6-1* complemented plants grown under ambient CO2 or (D) elevated (1000 ppm) CO2. Columns indicate mean values, error bars = s.e.m. (n=5). (E) Epidermal peels were taken from WT or *pme6-1* leaves and incubated for 2 hours in the light (300µmol m-2 s-1) in opening buffer supplied with CO2 free air before addition of ABA to 1 or 10µM, as indicated. Apertures were measured after incubation for a further 2 hours. Each column indicates the mean stomatal aperture achieved with error bars indicating s.e.m. A t-test was performed on each pair of measurements (WT vs *pme6-1*) with single asterisk (*) indicating a significant difference at p = 0.05 and double asterisk (**) indicating a significant difference at p = 0.01.

Table S1 Summary of primary antibodies used and the binding pattern observed.

Antibody	Cell wall epitope	Binding in mature leaves	References
	**Hemicelluloses**		
LM10	(1-4)-B-D-xylan	Yes: Vasculature	[S5]
LM11	(1-4)-B-D-xylan/arabinoxylan	Yes: Vasculature	
LM15	Xyloglucan (XXXG)	Yes: Guard cells and weakly in epidermal cells	[S6]
LM24	Xyloglucan	None	[S7]
LM25	Xyloglucan	None	
LM21	Mannan	Yes: Broad binding	[S8]
LM22	Mannan	None	
	**Pectins**		
JIM5	Partially methylated homogalacturonan	None	[S9]
JIM7	Partially/completely de-esterified homogalacturonan	Yes: Broad binding	
2F4	Calcium-crosslinked pectins	Yes: Guard cell junctions	[S10]
Pam1	Blockwise de-esterified homogalacturonan	None	[S11]
LM7	Non-blockwise de-esterified homogalacturonan	None	[S12]
LM18	Partially de-esterified HGA	Yes: Broad binding. Less binding in guard cells	[S9]
LM19	unesterified homogalacturonan	Yes: Broad binding	
LM20	highly esterified homogalacturonan	Yes: Yes, absent from guard cells	
LM8	Xylogalacturonan	None	[S13]
LM5	(1->4)-b-D-galactan	Yes: Epidermis, absent from guard cells	[S14]
LM9	Feryloylated (1->4)-b-D-galactan	None	[S15]
LM6	(1->5)-a-L-arabinan	None	[S16]
LM13	Linearised-(1->5)-a-L-arabinan	Yes: Weak binding, present in guard cell ledges	[S17]
LM16	Processed arabinan	None	[S18]
	**Other glycans**		
LM12	Feruloylated arabinosyl/galactosyl	Yes: Epidermal, guard cell and vasculature binding	[S7]
LM23	Xylogalacturonan	None	

	**Extensins**		
LM1	Extensin	None	[S19]
JIM11	Extensin	None	[S20]
JIM12	Extensin	None	
JIM19	Extensin	None	[S19]
JIM20	Extensin	None	[S20]
	AGP'S		
LM2	b-linked GlcA	Yes: Vasculature	[S20]
LM14	AGP glycan	Yes: Broad binding	[S17]
JIM4	AGP glycan	None	[S21]
JIM8	AGP glycan	None	[S22]
JIM13	AGP glycan	None	[S23]
JIM14	AGP glycan	None	
JIM15	AGP glycan	None	
JIM16	AGP glycan	Yes: Epidermal and vasculature, less binding in guard cells	
MAC207	AGP glycan	None	

Supplemental Experimental Procedures

Plant material

Seeds were surface sterilised in bleach diluted in water (1:5 v/v) containing 0.05% (v/v) Tween20 and stratified at 4°C for 7 days. Seeds were then transferred to square pots of 6 cm diameter and 8 cm height containing 1:3 mix of perlite:soil and transferred to a controlled environment chamber and grown with 12 h light (200 μmol m^{-2} s^{-1}) with 22°C day temperature, 16°C night temperature and 60% humidity. Plants used for immunolabelling were taken at 21 days, and, plants used for aperture analysis and gas exchange at 28 days after transfer to the growth chamber.

For creation of the *PME6* promoter GUS reporter line, a region approximately 1200 bp upstream of the ATG promoter translational start codon of *PME6* was amplified from genomic DNA with primers 5′-CACCTGGGATCCAAAATGATTG-3'5'- TGTGGGATATTGTTTTCTTAGGG -3′ and KOD DNA polymerase, inserted into the pENTR-D-TOPO entry vector (Invitrogen), and recombined with the pKGWFS7 destination vector [S1] before transfer into *Agrobacterium tumefaciensC58* cells and transformation into Col-0 Arabidopsis by floral dip [S2]. Seeds were selected on 50 μgml^{-1} kanamycin and insertion confirmed by PCR using forward $_{Pro}$PME6 specific primer and GUS gene reverse primer (5′-TGCTCAGGTAGTGGTTGTCG-3′).

The *pme-6* T-DNA insertion line (SGT6342) was obtained from NASC (Nottingham,UK) and confirmed as homozygous for the insertion by PCR using primers 5′-TCTGAGTCGTGTAAACGAGCC - and 5′-CCTCTTCGTATTCAAAGTATTTCCC. To create the *pme6-1* line complemented with *PME6* the coding region of *PME6* was amplified from the vector pUNi-At1g23200 (U18916;ABRC) with primers 5'-CACCAACCTAAACAAAAAAACC- and 5'-GATGACAACCGATTAAATTAATAAC and recombined into the pENTR-D-TOPO vector. This was then recombined by LR reaction into pMDC32 [S3] then excised with AscI. The pENTR-D-TOPO containing the *PME6* promoter (described above) was cut with AscI and the *PME6* coding region ligated 3' of the promoter. The plasmid was recombined by an LR reaction with pHGW [S1] to create the proPME6::PME6 construct before transfer into *Agrobacterium tumefaciensC58* cells and transformation into the *pme6-1* background by floral dip [S2]. The complemented *pme6-1* line is referred to as $_{pro}$PME6::PME6. Transformants were selected on 0.5X MS (Murashige and Skoog) medium, 1.5% (w/v) sucrose containing 15mgL^{-1} hygromycin and plants from the T3 generation analysed.

Gene expression analysis and immunolabelling

For analysis of *PME6* expression, RNA was extracted from seedlings using a Qiagen RNAeasy kit, and reverse transcribed into cDNA using oligo dT primer and SuperscriptII (Invitrogen). PCR was carried out on cDNA to determine if any transcript was detectable. Primer 5'-GGAAGATTCCAAAACTACGGC and 5'-GCCGTCCTAAATAAGTTTCCG were used to detect *PME-6* transcript, RUB1 (AT4G36800) primers were used as a positive control; (5'-GCGAACTTCGTCTTCACAA and 5'-GGAAAAAGGTCTGACCGACA.

Histochemical staining for GUS activity was carried out on leaves of T2 seedlings in 50 mM potassium phosphate, 1 mM potassium ferrocyanide, 1 mM potassium ferricyanide, 0.2% (v/v) Triton X-100, 2 mM 5-bromo-4-chloro-3-indolyl-β-d-glucuronic acid, and 10 mM EDTA after vacuum infiltration at 37°C. Leaves were decolorized overnight with 70% (v/v) ethanol, and washed in 10% glycerol. Images were captured with an Olympus BX51 microscope connected to a DP70 digital camera. Expression patterns shown were typical of several independently transformed lines.

For immunolabelling, 21-day-old leaf samples (3 mm squares) were fixed in 4% (w/v) formaldehyde in PEM buffer (0.1 M PIPES, 2 mM EGTA, 1 mM $MgSO_4$, adjusted to pH 7) by vacuum infiltration then dehydrated in an ethanol series (30 min each at 30%, 50%, 70%, 100%

EtOH) and infiltrated with LR White Resin (London Resin Company) diluted in ethanol (45 min each at 10%, 20%, 30%, 50%, 70% & 90% resin then 3x8 h at 100%). Samples were stood vertically in gelatine capsules filled with resin and allowed to polymerise for 7 days at 37°C. Sections were cut to a thickness of 2 µm using a Reichert-Jung Ultracut E ultramicrotome using a glass knife.

Sections were incubated with 3% (w/v) milk protein (Marvel, Premier Beverages, UK) in phosphate-buffered saline solution (PBS, pH 7.2) (hereafter known as PBS/MP). Sections were then incubated with a ten-fold dilution of primary monoclonal antibody in PBS/MP for 1 h at room temperature. Samples were washed 3 times with PBS and secondary antibody was added (100-fold dilution in PBS/MP) for 1 h. Samples were kept in the dark from this step. For the JIM- and LM- series of antibodies anti-rat-IgG (whole molecule) coupled to fluorescein isothiocyanate (FITC) was used, for the 2F4 antibody, an anti-mouse-IgG (whole molecule) coupled to FITC was used. Samples were counterstained with 0.25% (w/v) Calcofluor White solution diluted ten-fold in PBS for 5 min before mounting on slides with Citifluor AF1 anti-fade solution (Agar Scientific, UK). Samples were visualised on an Olympus BX51 microscope with epifluorescence optics fitted and images captured using a DP51 camera. FITC was visualised using a filter set with 460-490 nm excitation filter, a 510-550 nm emission filter and a 505 nm dichroic mirror. Calcofluor White was visualised using a 400-410 nm excitation filter, a 455 nm emission filter and a 455 nm dichroic mirror.

The reproducibility of antibody patterns were assessed by a scoring technique. 50 stomata were assessed and the pattern of immunolabelling was classed in terms of its prevalence in the guard cells. Guard cells which were fully labelled with antibody, as typified by JIM7 labelling were classed as “Fully” labelled, guard cells which had some signal in the guard cell but not distributed throughout the whole cell were classed as “partial” and stomata which had no labelling inside the guard cell but did show signal at the junctions between guard cells and their neighbouring cells were classed as “Junctions only”. No stomata analysed fell outside of these three categories.

Electron Microscopy

For cryo-scanning electron microscopy (cryo-SEM), leaves were careful removed with forceps and placed flat on a brass stub, stuck down with a cryo glue preparation consisting of a 3:1 mixure of Tissue-Tec (Scigen Scientific, USA)) and Aquadag colloidal graphite (Agar Scientific, Stansted, UK)) and then plunge frozen in liquid nitrogen with vacuum applied. For sample preparation for cryo fracture, leaves were placed vertically in recessed stubs held by the cryo glue preparation. Frozen samples were then transferred under vacuum to the prep chamber of a PT3010T cryo-apparatus (Quorum Technologies, Lewes, UK) and maintained at -145C. Surface ice was removed using a sublimation protocol consisting of -90C for 3 min. For cryofracture, no sublimation was carried out and instead a level semi-rotary cryo knife was used to randomly fracture the leaf. All samples were sputter coated with platinum until a measured thickness of 5 nm was recorded. Samples werethen transferred and maintained cold, under vacuum into the chamber of a Zeiss EVO HD15 SEM fitted with a cryo-stage. Images were taken on the SEM using a gun voltage of 6 kV, I probe size of 460 pA, a SE detector and a working distance of between 5 and 6mm. For transmission electron microscopy, leaves were dissected into 3% (w/v) glutaraldehyde (Sigma-Aldrich) in 0.1 M phosphate buffer. Further fixation and processing were as described previously [S4]

Stomatal aperture measurements

Abaxial epidermal peels of mature leaves were removed at least 2 hours into the photoperiod and floated onto opening buffer (10 mM KCl, 10 mM MES, pH 6.2). Samples were maintained at 22°C with 200 µmol $m^{-2} s^{-1}$ of light. For CO_2 responses air was bubbled into the opening buffer containing either 0 ppm CO_2 (CO_2 free treatment), ambient CO_2, or 1000ppm CO_2. For mannitol response

samples, 0.5 M mannitol was bubbled into the opening buffer. For ABA responses epidermal peels were incubated in opening buffer supplied with CO_2 free air for two hours before ABA was added to the buffer. For mannitol responses peels were incubated in 0.5M mannitol added to buffer (10 mM MES, pH6.2). Epidermal peels were imaged after 2 hours using an Olympus BX51 microscope and DP70 digital camera and stomatal apertures measured. 40 stomatal apertures were measured for each treatment in each of three independent experiments. For each experiment epidermal peels were taken from at least 3 plants of each genotype.

Thermal imaging

Infrared images were taken using a FLIR SC660 camera (FLIR systems). The camera was positioned 1 m above the leaf rosette. Plants were imaged at 24-days old under well-watered conditions at which point water was withheld. Plants were then imaged again at 29 days under strong drought conditions. 6 plants of each genotype were imaged and subsequent analysis was conducted using ThermaCAM researcher v2.10 professional (FLIR systems).

Gas exchange analysis

CO_2 shifts were conducted on 28-day old plants using mature non-senescent leaves. Analysis was started 2 hours into the photoperiod of the growth chamber and did not continue into the last 3 hours of the photoperiod. Measurements were taken using a LI-6400 infrared gas exchange analyser system using a leaf fluorometer chamber (LI-COR Inc.) with a $2cm^2$ circular area for measurement. Temperature was held at 21°C and humidity was kept above 58% and below 65%. Photon flux density was held at 300 µmol m^{-2} s^{-1} with 10% blue light. In cases where the leaf did not fill the chamber, leaf area was measured and a correction made in subsequent analysis. To assess stomatal response to CO_2 conductance was stabilised at 500 ppm for 40 minutes, CO_2 was then shifted to 1000 ppm for 50 minutes to stimulate stomatal closing, and then to 100ppm for 50 minutes to stimulate stomatal closure. A/Ci response curves were measured on young fully expanded leaves at 21°C leaf temperature, 1200 µmol m^{-2} s^{-1} PPFD light, and approximately 60% relative humidity. Once leaves were acclimated to chamber conditions, measurements were taken at 400, 250, 150, 100, 80, 60, and 40 every 2-3 minutes at 200 µmol s^{-1} flow rate, then at 400, 500, 600, 800, 900, 1000, 1200, 1400, and 1600 every 3-5 minutes at 300 µmol s^{-1} flow rate.

Analysis of stomatal size and density

For stomatal density analysis fully expanded non-senescent leaves were harvested from 35 day old seedlings. Leaves were fixed in 4% (v/v) formaldehyde in PEM buffer (0.1 M PIPES, 2 mM EGTA, 1 mM $MgSO_4$, adjusted to pH 7) for 8 hours. Leaves were then washed twice in 70% (v/v) ethanol for 30 minutes each wash. Tissue was then cleared by incubation in chloral hydrate (2.5 g mL-1) in 30% (v/v) glycerol twice for 8 h. Samples were then mounted in 30% (v/v) glycerol solution and imaged on an Olympus BX51 microscope under the 40x objective using Nomarsky illumination, images were captured with an Olympus DP70 camera and the number of stomata counted. 4 viewpoints per leaf were analysed and 3 leaves per plant.

For stomatal size analysis abaxial epidermal peels were taken and floated onto opening buffer ((10 mM KCl, 10 mM MES, pH 6.2). Samples were maintained at 22°C with 200µmol of light. CO_2 free air was bubbled through the buffer to promote stomatal opening. Epidermal peels were imaged after 2 hours using an Olympus BX51 microscope with a DP70 digital camera and stomatal complex length was measured.

Analysis of rosette area

Mature Arabidopsis plants were photographed at 30 days old from a height of 30cm using and Olympus E-PL1 digital camera. Rosette area was measured in ImageJ using the colour threshold tool to isolate the rosette in the image.

Supplemental references

S1. Karimi, M., Depicker, A., and Hilson, P. (2007). Recombinational cloning with plant gateway vectors. Plant Physiol. *145*, 1144–1154.

S2. Clough, S. J., and Bent, A. F. (1998). Floral dip: A simplified method for Agrobacterium-mediated transformation of Arabidopsis thaliana. Plant J. *16*, 735–743.

S3. Brand, L., Hörler, M., Nüesch, E., Vassalli, S., Barrell, P., Yang, W., Jefferson, R. a, Grossniklaus, U., and Curtis, M. D. (2006). A versatile and reliable two-component system for tissue-specific gene induction in Arabidopsis. Plant Physiol. *141*, 1194–1204.

S4. Wallace, S., Chater, C. C., Kamisugi, Y., Cuming, A. C., Wellman, C. H., Beerling, D. J., and Fleming, A. J. (2015). Conservation of Male Sterility 2 function during spore and pollen wall development supports an evolutionarily early recruitment of a core component in the sporopollenin biosynthetic pathway. New Phytol. *205*, 390–401.

S5. McCartney, L., Marcus, S. E., and Knox, J. P. (2005). Monoclonal Antibodies to Plant Cell Wall Xylans and Arabinoxylans\r10.1369/jhc.4B6578.2005. J. Histochem. Cytochem. *53*, 543–546.

S6. Marcus, S. E., Verhertbruggen, Y., Hervé, C., Ordaz-Ortiz, J. J., Farkas, V., Pedersen, H. L., Willats, W. G. T., and Knox, J. P. (2008). Pectic homogalacturonan masks abundant sets of xyloglucan epitopes in plant cell walls. BMC Plant Biol. *8*, 60.

S7. Pedersen, H. L., Fangel, J. U., McCleary, B., Ruzanski, C., Rydahl, M. G., Ralet, M. C., Farkas, V., Von Schantz, L., Marcus, S. E., Andersen, M. C. F., et al. (2012). Versatile high resolution oligosaccharide microarrays for plant glycobiology and cell wall research. J. Biol. Chem. *287*, 39429–39438.

S8. Marcus, S. E., Blake, A. W., Benians, T. A. S., Lee, K. J. D., Poyser, C., Donaldson, L., Leroux, O., Rogowski, A., Petersen, H. L., Boraston, A., et al. (2010). Restricted access of proteins to mannan polysaccharides in intact plant cell walls. Plant J. *64*, 191–203.

S9. Verhertbruggen, Y., Marcus, S. E., Haeger, A., Ordaz-Ortiz, J. J., and Knox, J. P. (2009). An extended set of monoclonal antibodies to pectic homogalacturonan. Carbohydr. Res. *344*, 1858–62.

S10. Liners, F., and Van Cutsem, P. (1992). Distribution of pectic polysaccharides throughout walls of suspension-cultured carrot cells - An immunocytochemical study. Protoplasma *170*, 10–21.

S11. Willats, W. G. T., Limberg, G., Buchholt, H. C., Van Alebeek, G. J., Benen, J., Christensen, T. M. I. E., Visser, J., Voragen, A., Mikkelsen, J. D., and Knox, J. P. (2000). Analysis of pectic epitopes recognised by hybridoma and phage display monoclonal antibodies using defined oligosaccharides, polysaccharides, and enzymatic degradation. Carbohydr. Res. *327*, 309–320.

S12. Willats, W. G. T., Orfila, C., Limberg, G., Buchholt, H. C., Van Alebeek, G. J. W. M., Voragen, A. G. J., Marcus, S. E., Christensen, T. M. I. E., Mikkelsen, J. D., Murray, B. S., et al. (2001). Modulation of the degree and pattern of methyl-esterification of pectic homogalacturonan in plant cell walls: Implications for pectin methyl esterase action, matrix properties, and cell adhesion. J. Biol. Chem. *276*, 19404–19413.

S13. Willats, W. G. T., McCartney, L., Steele-King, C. G., Marcus, S. E., Mort, A., Huisman, M., Van Alebeek, G. J., Schols, H. A., Voragen, A. G. J., Le Goff, A., et al. (2004). A xylogalacturonan epitope is specifically associated with plant cell detachment. Planta *218*, 673–681.

S14. Jones, L., Seymour, G. B., and Knox, J. P. (1997). Localization of Pectic Galactan in Tomato Cell Walls Using a Monoclonal Antibody Specific to (1[-]4)-[beta]-D-Galactan. Plant Physiol. *113*, 1405–1412.

S15. Clausen, M. H., Ralet, M.-C., Willats, W. G. T., McCartney, L., Marcus, S. E., Thibault, J.-F., and Knox, J. P. (2004). A monoclonal antibody to feruloylated-(1-->4)-beta-D-galactan. Planta *219*, 1036–41.

S16. Willats, W. G. T., Marcus, S. E., and Knox, J. P. (1998). Generation of a monoclonal antibody specific to (1→5)-α-l-arabinan. Carbohydr. Res. *308*, 149–152.

S17. Moller, I., Marcus, S. E., Haeger, A., Verhertbruggen, Y., Verhoef, R., Schols, H., Ulvskov, P., Mikkelsen, J. D., Knox, J. P., and Willats, W. (2008). High-throughput screening of monoclonal antibodies against plant cell wall glycans by hierarchical clustering of their carbohydrate microarray binding profiles. Glycoconj. J. *25*, 37–48.

S18. Verhertbruggen, Y., Marcus, S. E., Haeger, A., Verhoef, R., Schols, H. A., McCleary, B. V., McKee, L., Gilbert, H. J., and Knox, J. P. (2009). Developmental complexity of arabinan polysaccharides and their processing in plant cell walls. Plant J. *59*, 413–425.

S19. Smallwood, M., Martin, H., and Knox, J. P. (1995). An epitope of rice threonine- and hydroxyproline-rich glycoprotein is common to cell wall and hydrophobic plasma-membrane glycoproteins. Planta An Int. J. Plant Biol. *196*, 510–522.

S20. Smallwood, M., Beven, A., Donovan, N., Neill, S. J., Peart, J., Roberts, K., and Knox, J. P. (1994). Localization of cell wall proteins in relation to the developmental anatomy of the carrot root apex. Plant J. *5*, 237–246.

S21. Knox, J. P., Day, S., and Roberts, K. (1989). A set of cell surface glycoproteins forms an early marker of cell position , but not cell type , in the root apical meristem of Daucus carota L . Development *56*, 47–56.

S22. Pennell, R., Janniche, L., Kjellbom, P., Scofield, G., Peart, J., and Roberts, K. (1991). Developmental Regulation of a Plasma Membrane Arabinogalactan Protein Epitope in Oilseed Rape Flowers. Plant Cell *3*, 1317–1326.

S23. Yates, E. A., and Knox, J. P. (1994). Investigations into the occurrence of plant cell surface epitopes in exudate gums. Carbohydr. Polym. *24*, 281–286.
